# Computational Account of Spontaneous Activity as a Signature of Predictive Coding

**DOI:** 10.1371/journal.pcbi.1005355

**Published:** 2017-01-23

**Authors:** Veronika Koren, Sophie Denève

**Affiliations:** 1 Group for Neural Theory, Département d’Études Cognitives, École Normale Supérieure, Paris, France; 2 Neural Information Processing Group, Institute of Software Engineering and Theoretical Computer Science, Technische Universität Berlin, Berlin, Germany; 3 Bernstein Center for Computational Neuroscience Berlin, Berlin, Germany; George Mason University, UNITED STATES

## Abstract

Spontaneous activity is commonly observed in a variety of cortical states. Experimental evidence suggested that neural assemblies undergo slow oscillations with Up ad Down states even when the network is isolated from the rest of the brain. Here we show that these spontaneous events can be generated by the recurrent connections within the network and understood as signatures of neural circuits that are correcting their internal representation. A noiseless spiking neural network can represent its input signals most accurately when excitatory and inhibitory currents are as strong and as tightly balanced as possible. However, in the presence of realistic neural noise and synaptic delays, this may result in prohibitively large spike counts. An optimal working regime can be found by considering terms that control firing rates in the objective function from which the network is derived and then minimizing simultaneously the coding error and the cost of neural activity. In biological terms, this is equivalent to tuning neural thresholds and after-spike hyperpolarization. In suboptimal working regimes, we observe spontaneous activity even in the absence of feed-forward inputs. In an all-to-all randomly connected network, the entire population is involved in Up states. In spatially organized networks with local connectivity, Up states spread through local connections between neurons of similar selectivity and take the form of a traveling wave. Up states are observed for a wide range of parameters and have similar statistical properties in both active and quiescent state. In the optimal working regime, Up states are vanishing, leaving place to asynchronous activity, suggesting that this working regime is a signature of maximally efficient coding. Although they result in a massive increase in the firing activity, the read-out of spontaneous Up states is in fact orthogonal to the stimulus representation, therefore interfering minimally with the network function.

## Author Summary

Spontaneous bursts of activity, commonly observed in the brain, can be understood in terms of error-correcting computation within a neural network. Bursts arise automatically in a network that is inefficiently correcting its internal representation.

## Introduction

A growing amount of experimental evidence suggests a complex interaction between stimulus-driven and spontaneous activity [1–3]. In sensory cortices of awake behaving animals, neural activity is present both during periods when the neural population is driven by sensory stimuli, as well as in absence of those. We refer to working regimes in the presence of stimuli as active states and to the working regimes in the absence of external drive as quiescent states (Table 1). In the absence of external drive, the population activity can take the form of characteristic synchronized bursts of spiking activity, or Up states, interspersed by periods of silence or Down states [1]. Stimulus-driven and spontaneous activity are difficult to distinguish from one another [1]. Spontaneous Up states in a variety of cortical states share many of the statistical properties of stimulus-driven spiking responses [4].

**Table 1:** Terminology.

Phenomenon	Description
Active state	working regime in the presence of external drive
Quiescent state	working regime in the absence of external drive
Up state	burst of population activity in either active or quiescent state
Down state	absence of activity in quiescent state

Why does spontaneous activity occur and what could be its role in terms of computation? This phenomenon could be understood mechanistically as arising in a recurrently connected network. For example, it could be triggered by bottom-up pathways recruiting a dynamic interplay of local excitation, adaptation and inhibition [5, 6]. Alternatively, up-states could be caused by modulations from distal areas, either top-down or sub-cortical, like a “gate” closing or opening to let sensory information in [7]. Finally, Up states could also arise within the cortical network. In fact, electrophysiology in slices has shown that cortical microcircuits, separated from the rest of the brain, spontaneously oscillate between Up and Down states [8]. Intrinsically generated slow oscillations have also been replicated with a biophysical model network [9]. To this day, however, few studies have investigated what could be the computational role of such striking phenomena. Here we propose a simple functional account of spontaneous activity as a signature of predictive coding. More precisely, spontaneous bursts of population activity could be caused by the network that is automatically correcting itself after inadvertently responding to noise.

Cortical circuits maintain a tight balance between excitation and inhibition, which can account for the large variability of neural spike trains [10–12]. The balance of excitation and inhibition persists even during spontaneous Up states [13]. It was shown previously that E/I balance implements a form of predictive coding: by maintaining a tight balance between its feed-forward and recurrent inputs, a population of neurons monitors and automatically corrects its own coding errors. Any coding error induces additional spikes, recruits more inhibition and automatically restores the balance [14] (see also methods, section 1.1). In a low-noise scenario and with instantaneous synapses, this self-correction results in a population code that is very precise and parsimonious with spikes. The read-out of such code is almost deterministic, contrasting with the large variability it creates in single neuron’s responses. However, in the presence of realistic noise and synaptic delays, such network could constantly spike to correct its own mistakes, resulting in very large firing rates. A similar issue arises when there are synaptic delays. Several neurons can be recruited simultaneously before they had time to inhibit each-other, resulting in synchronization that can severely degrade the coding efficiency. In such scenario, some amount of noise is actually required to limit the detrimental effects of oscillations on coding precision [16–18].

In order to limit inefficient spiking in noisy and delayed networks, one can rely on homeostatic cost, penalizing strong firing rates. This penalty term has simple biophysical interpretation as it consists in increasing neural threshold potentials and in strengthening the hyper-polarization of the neural membrane after a spike. Note that all of those are not simply added to the network but follow from the derivation of an objective function (see methods, section 1.2). All terms derived from the cost terms have the effect of controlling the “readiness for spiking” or, in more biological terms, the excitability of the network. For a given level of noise and synaptic delays, we find that there is a clearly defined “sweet spot” for cost parameters. With an optimal cost, the network is maximally efficient—it is both accurate in representing its input signals and parsimonious with spikes. For costs lower than optimal, the network regularly enters into epochs of highly synchronized firing. The activity alternates between rare and transient Up states, i.e. periods of synchronized activity with high firing rates, and longer Down states, periods with low firing rates and asynchronous firing. For costs stronger than optimal, coding error progressively increases. In the optimal regime, coding error is minimal. Interestingly, this regime is also the one where Up states vanish. The range over which coding is highly efficient therefore strongly depends on the dynamical regime in which the network operates. The coincidence of the optimal coding regime and the transition to asynchronous dynamical state is robust to the particular choice of network parameters as well as the level of noise, suggesting that this phenomenon is a general property of a spiking network with predictive coding.

Our approach is novel in that it suggests a direct functional relationship between stimulus-driven and spontaneous activity. The model can account for the continuum of working regimes (or states) observed in biological networks, with two parameters representing the weighting of the accuracy of signal representation over cost on spiking. In particular, we propose that quiescent state activity can be understood as a special case of evoked activity, since same computational rules govern the behavior of the network in both cases. Spontaneous Up states occur when the network maintains itself in a suboptimal state for encoding stimuli and can be understood as a lack of homeostatic balance in a biological network. We first describe simple toy examples that capture these basic phenomena before switching to small microcircuits with all-to-all connections and transmission delays. Finally, we present networks with spatial organization and local connectivity. We show that same computational principles generate Up states in all cases.

## Methods

### 1 Efficient coding

#### 1.1 Efficient coding without transmission delays

The model with predictive coding is derived from minimization of an objective function with spikes [14]. Consider a population of neurons receiving a set of inputs *s*_*j*_(*t*), *j* = 1, 2, …*J*, identical for all neurons. The current resulting from feed-forward connections in the neuron’s membrane potential is a weighted sum of inputs, ∑_*j*_ *w*_*ij*_*s*_*j*_(*t*), where *w*_*ij*_ is the weight of neuron *i* for the input *s*_*j*_. The output spikes of the network are decoded as a weighted sum of neural firing rates,

$$\hat{x_{j}}\left(t\right)=\sum_{i=1}^{N}w_{ij}r_{i}\left(t\right)$$ (1)

with *N* the number of neurons. *r*_*i*_(*t*) is the instantaneous firing rate of neuron *i*. The instantaneous firing rate is defined as the convolution of the spike train with an exponential filter, *u*(*t*) = exp(−*λt*)

$$r_{i}\left(t\right)=\int_{0}^{\infty }u\left(\tau \right)o_{i}\left(t-\tau \right)d\tau$$ (2)

where *o*_*i*_(*t*) is the spike train of neuron *i*, $o_{i}\left(t\right)=\sum_{k}\delta \left(t-t_{i}^{k}\right)$, *δ*(*t*) is the Dirac delta function and $t_{i}^{k}$ is the k-th spike of the neuron *i*. Exponential filter captures short-lasting causal effects of biological variables. Note that the feed-forward input and the neuron’s contribution to the decoded signal are weighted by the same amount, *w*_*ij*_. This is a reasonable assumption, considering that neurons with stronger synapses will also send bigger PSPs to other neurons and at the same time contribute more to the read-out of the estimated signal. In addition to biophysically well defined variables cited above, we define an abstract variable, the signal, which is the convolution of the input with the same temporal filter as the decoder.

$$x_{j}\left(t\right)=\int_{0}^{\infty }u\left(t^{\prime }\right)s_{j}\left(t-t^{\prime }\right)dt^{\prime }$$ (3)

It is important to remark the difference between the input, which is the current that strikes the neural membrane, weighted by neuron’s weight according to the strength of its synapses, and the signal, which is what the network is trying to represent and approximate with its activity. The neural membrane has capacitive properties which results in temporal filtering of its inputs. If we assume that neuron’s membrane potential is a leaky integrator of an input *s*(*t*), we can write its sub-threshold dynamics as $V\left(t\right)=\int_{0}^{\infty }u\left(t^{\prime }\right)s\left(t-t^{\prime }\right)dt^{\prime }$, which is equivalent to the expression for *x*_*j*_(*t*). The input *s*(*t*) and the signal *x*(*t*) therefore cannot be the same variable.

When appropriate, we will use vector notation and refer to the variable **x**(*t*) = [*x*_1_(*t*), *x*_2_(*t*), …, *x*_*J*_(*t*)] as the signal and to the variable $\hat{x}\left(t\right)=\left[{\hat{x}}_{1}\left(t\right),{\hat{x}}_{2}\left(t\right),...,{\hat{x}}_{J}\left(t\right)\right]$ as the decoded signal or the estimate of the signal. Vector notation will also be used when referring to the complete set of weights, **w** = [*w*_11_, *w*_12_, …*w*_1*N*_; …;*w*_*J*1_, * w*_*J*2_, …*w*_*JN*_], where *N* is the number of neurons in the network and *J* is the number of inputs. In the reference paper [14], neurons have slow and fast currents and the emergence of slow currents is due to assuming different temporal filters for the signal and for the decoder. With that assumption, there is no leak current that follows from derivation, the later being added to the model for biological plausibility. In the present work we pose those filters as identical, which results in a network with fast currents only and where the leak current follows from the derivation of the model. This procedure is similar to the one used in [15], but with cost parameters that are added to the objective function.

The functional goal of an efficient network is to track an arbitrary signal with maximal efficiency (i.e. with best accuracy and with minimal number of spikes). In [14], the model is named “network with predictive coding”, where the term “predictive coding” refers to the fact that spikes arise when there is a prediction error. The term “prediction” should not be understood as “looking into the future” but rather as having a signal, which is partially predicted by the current read-out. This is achieved by posing as objective the minimization of the following cost function:

$$E\left(t\right)=∥x\left(t\right)-\hat{x}{\left(t\right)∥}^{2}$$ (4)

and assuming there will be a spike only when this minimizes this function, i.e.

E(t|neuronispikes)<E(t,nospike). (5)

The objective function evaluates the distance between the signal and its estimate in real time and is therefore a time-dependent variable. This is motivated by the fact that biological neurons receive inputs and generate output spikes in real time. We assume that the minimization of the objective function with spikes in real time is a computational problem that is biologically plausible. The objective function is minimized for the next time step only (greedy minimization), by assuming stationarity of the stimulus between the present and the next time step. From the minimization of the objective function, the condition for spiking is derived (see [14]):

$$w_{i}^{T}\left(x\left(t\right)-\hat{x}\left(t\right)\right)>\sum_{j=1}^{J}\frac{w_{ij}^{2}}{2}.$$ (6)

Assuming the left-hand side is equivalent to the membrane potential of a single neuron,

$$V_{i}\left(t\right)=w_{i}^{T}\left(x\left(t\right)-\hat{x}\left(t\right)\right)$$ (7)

and the right-hand side to the firing threshold, we get neurons that track the stimulus with great precision, as any error bigger than the half of the weight of a single neuron will trigger error-correcting activity. The assumption that eq 7 is the membrane potential is reasonable if one remembers that the condition in eq 5 was a condition for having a spike and that a spike is fired when the membrane potential reaches the firing threshold.

When the condition for spiking is derived with respect to time, we get the following expression:

$${\dot{V}}_{i}\left(t\right)=-\lambda V_{i}\left(t\right)+\sum_{j}w_{ij}s_{j}\left(t\right)-\sum_{k}\phi_{ik}o_{k}\left(t\right)$$ (8)

where *ϕ* = **w**^**T**^ **w** is the matrix of lateral and recurrent connections. Note that eq 8 is a Leaky Integrate-and-fire neuron with leak term, feed-forward current and a current that results from lateral (between neurons) and recurrent (autapse) connections.

To understand better how the model works, we present the simplest case, a single neuron tracking one input (“auto-encoder”). We assign to the neuron a weight, *w* = 1. Here, predictive coding corresponds to a simple reset mechanism, computationally equivalent to self connection with weight -1. The membrane potential is equivalent to the prediction error, $V\left(t\right)=x\left(t\right)-\hat{x}\left(t\right)$. Whenever it crosses the threshold ($Thres=\frac{w^{2}}{2}=\frac{1}{2}$), the neuron fires a spike. At this time, the autapse of the neuron activates and resets the membrane potential of the neuron for −*w* = −1. Notice that there is no need to implement the reset artificially. Minimization of the objective function in the one neuron example is equivalent to the firing rule $V\left(t\right)>\frac{1}{2}$, where the membrane potential is integrated according to the simple rule, $\dot{V}=-\lambda V\left(t\right)+s\left(t\right)-o\left(t\right)$. Same model can be extended to an all-to-all connected network, receiving multiple inputs. The “auto-encoding” is now performed via lateral connections that are canceling the feed-forward input. Currents resulting from feed-forward as well as from lateral connections can be either inhibitory or excitatory. Interestingly, the error signal is now common to all the neurons in the network, since the error correcting spikes are communicated to other neurons via lateral connections. This results in a population code where all neurons in the network jointly encode the incoming signals.

#### 1.2 Efficient coding with transmission delays

Without transmission delays, the network described by eq 8 is optimal in tracking an arbitrary multidimensional signal. However, biological neurons have transmission delays. It turns out that having transmission delays has a great impact on network dynamics as well as on the accuracy with which signals are encoded. From the dynamics point of view, we observe massive synchronization of neurons with similar selectivity, which naturally deteriorates coding efficiency. Transmission delays cause the delay in the error signal, since the latter depends on the read-out of network activity. All neurons receive a delayed error signal and since they do not have the information about the present state in other neurons, all neurons whose spike will contribute to the minimization of the error will spike synchronously. This results in alternative spiking of neurons with + and - selectivity, where + and − neurons tend to synchronize.

The overall amount of synchronization can be controlled by imposing a “cost” on spiking. As in the reference work [14], we include a linear and a quadratic cost term, penalizing large spike counts. The objective function with cost terms is defined as follows:

$$E\left(t\right)=∥x\left(t\right)-\hat{x}{\left(t\right)∥}^{2}+\nu \sum_{i}r_{i}\left(t\right)+\mu \sum_{i}r_{i}{\left(t\right)}^{2}$$ (9)

Deriving the objective function with cost terms introduces an additional term in the membrane potential (see the last term on the right side):

$$V_{i}\left(t\right)=w_{i}^{T}\left(x\left(t\right)-\hat{x}\left(t\right)\right)-\mu r_{i}\left(t\right).$$ (10)

In [14], different time constants for the signal and the estimate are used. Here, we pose those time constants are the same and derive the membrane potential equation that we use for simulations. For easier reading, we will use vector notation. We define the vector of membrane potentials as follows: **V**(*t*) = *V*_1_(*t*), *V*_2_(*t*), …, *V*_*N*_(*t*), where *N* is the number of neurons. Similarly, we define a vector of firing rates and a vector of spike trains, **r**(*t*) = *r*_1_(*t*), *r*_2_(*t*), …, *r*_*N*_(*t*), **o**(*t*) = *o*_1_(*t*), *o*_2_(*t*), …, *o*_*N*_(*t*). Rewriting the eq 10 in vector notation gives the following:

$$V\left(t\right)=w^{T}\left(x\left(t\right)-\hat{x}\left(t\right)\right)-\mu r\left(t\right)$$ (11)

The derivative of the signal is defined as a leaky integration of inputs.

$$\dot{x}\left(t\right)=-\lambda x\left(t\right)+s\left(t\right)$$ (12)

The derivative of the instantaneous firing rate is defined as a leaky integration of spikes.

$$\dot{r}\left(t\right)=-\lambda r\left(t\right)+o\left(t\right)$$ (13)

Similarly, the derivative of the estimate is defined as a leaky integration of spikes, weighted by the weight matrix.

$$\dot{\hat{x}}\left(t\right)=-\lambda \hat{x}\left(t\right)+wo\left(t\right)$$ (14)

The derivative of the membrane potential is therefore the following:

$$\begin{array}{ccc} \dot{V}\left(t\right) & = & w^{T}\left(\dot{x}\left(t\right)-\dot{\hat{x}}\left(t\right)\right)-\mu \dot{r}\left(t\right)\\ & = & w^{T}\left(-\lambda x\left(t\right)+s\left(t\right)+\lambda \hat{x}\left(t\right)-wo\left(t\right)\right)-\mu \left(o\left(t\right)-\lambda r\left(t\right)\right)\\ & = & -\lambda \left(w^{T}\left(x\left(t\right)-\hat{x}\left(t\right)\right)-\mu r\left(t\right)\right)+w^{T}s\left(t\right)-w^{T}w\;o\left(t\right)-\mu o\left(t\right) \end{array}$$

Noticing that the expression in parenthesis is equivalent to the definition of the membrane potential (eq 11), we gain the leak term and get the expression for the membrane potential:

$$\dot{V}\left(t\right)=-\lambda V\left(t\right)+w^{T}s\left(t\right)-w^{T}w\;o\left(t\right)-\mu o\left(t\right)$$ (15)

For the single neuron, this gives the following:

$${\dot{V}}_{i}\left(t\right)=-\lambda V_{i}\left(t\right)+\sum_{j}w_{ij}s_{j}\left(t\right)-\sum_{k}\phi_{ik}o_{k}\left(t\right)-\mu o_{i}\left(t\right)+\sigma \eta_{i}\left(t\right)$$ (16)

where *ϕ* = **w**^**T**^ **w** is the matrix of lateral and recurrent connections. Derivation of the objective function with cost terms gives the following rule for firing:

$$V_{i}\left(t\right)>\sum_{j=1}^{J}\frac{w_{ij}^{2}}{2}+\frac{\mu }{2}+\frac{\nu }{2}$$ (17)

The effect of the linear cost *ν* is to raise thresholds of all neurons for the same amount and therefore to penalize high firing rates of the population (eq 17). The quadratic cost *μ* has the same network-wide effect on thresholds (eq 17) and in addition adds a hyperpolarizing current to the membrane potential (eq 16), only to the neuron that recently spiked. We interpret the latter as the spike-triggered adaptation. Finally, a noise term is added, which is a white noise with zero mean and standard deviation *σ*. Noise processes across neurons are uncorrelated, i.e. < *η*_*i*_(*t*)*η*_*j*_(*t*′) > = *δ*_*ij*_*δ*(*t* − *t*′).

### 2 Spiking in active and quiescent state: A minimal model

In general, costs on spiking are required to achieve efficient coding in networks with delays in synaptic transmission. In case costs on spiking are not properly controlling the activity, we observe a peculiar phenomenon which consists in fast oscillation of the read-out of neural activity. The fast oscillation is caused by alternative spiking of neurons with positive and negative selectivity, which we call “the ping-pong effect”. In case of the network with delays, the ping-pong appears even without any noise and is therefore due to transmission delays. In the network without transmission delays and without noise, there is no such effect, but it appears if the network gets perturbed by the noise. The network that is not regulated by costs on spiking is highly unstable and quickly enters inefficient coding regimes with excessively high firing rates. However, in the presence of noise, not all spikes are inefficient. If there is a spike that is only due to the noise, another spike of a neuron with opposite selectivity is in fact best suited to quickly correct the coding error. In the present section we illustrate efficient and inefficient spikes with a minimal model with 2 neurons and the linear cost, where spikes can be followed step-by-step.

In the case of a single neuron, Up states are not observed. However, encoding a signal that can be both positive and negative with only one neuron results in poor estimation of the signal. A single neuron is only able to correct the estimate in the direction of its weight. Consider the case where the single neuron has a positive weight. When the estimate is too small with respect to the signal, this neuron fires spikes, which brings the estimate closer to the signal. However, when the estimate is too large with respect to the signal, this neuron cannot correct for it and the error accumulates. Minimal models with one input and with 2 neurons, one with positive and one with negative weight, will be considered. This case is not biologically realistic but is a toy example, useful to grasp the full model. With the toy model in mind, one can extend it to more complex cases of multiple neurons and a high-dimensional signal.

#### 2.1 Minimal model for tracking the signal

In the minimal model, we have two neurons with arbitrary + and - weights, **w** = (*w*_1_, *w*_2_) = (*a*, −*b*), *a*, *b* > 0. Firing thresholds for the two neurons are proportional to their respective weights, ${\text{Thres}}_{1}=\frac{a^{2}}{2}+\frac{\nu }{2}$, ${\text{Thres}}_{2}=\frac{b^{2}}{2}+\frac{\nu }{2}$. The connectivity matrix is the following: $w^{T}w=\{\begin{array}{cc} a^{2} & -ab\\ -ab & b^{2} \end{array}\}$, and since the interaction term is −**w**^*T*^ **w**, the two neurons are connected to each other with symmetric excitatory connection (off-diagonal elements) and have an inhibitory autapse proportional to their respective weights (diagonal elements). Such a pair of neurons with opposite selectivity constitutes the minimal network that is successful in tracking even a fast signal, albeit with high firing rates. The two neurons are receiving the same input, but since they are weighting this input with + (neuron 1) and − (neuron 2) weights, their feed-forward currents are of opposite sign (the feed-forward current is *w*_*i*_*s*(*t*)). Note that neuron’s weight is in fact neuron’s selectivity; not for the input itself, but for the internal signal that the network as a whole needs to estimate.

#### 2.2 Error-correcting spiking in the quiescent state

In the quiescent state, there is no feed-forward input to the network (*s*(*t*) = 0 ∀*t*) and the signal remains silent at all times ($\bar{s}\left(t\right)=0\;\forall t$). Since we are most interested in activity that arises from the network alone, we will assume that neuron 1 is perturbed by the noise, sufficiently strong to provoke one single spike, and that after the first spike there is no more noise in the system. At the moment of perturbation, we assume that *V*_2_ is at the resting potential, *V*_2_ = 0. Before the first spike, firing rates are at zero and the estimated signal is silent, $\hat{s}\left(t\right)=0$. Firing rule reduces to the following:$-w_{i}^{T}\hat{s}\left(t\right))+\sigma \int_{t^{\prime }}\eta_{i}\left(t\right)dt>\frac{w_{i}^{2}}{2}+\frac{\nu }{2}.$ To initiate spiking, integration of the noise alone has to lead to crossing of the threshold:

$$\sigma \int_{0}^{t^{\prime }-dt}\eta_{1}\left(t\right)dt>\frac{w_{1}^{2}}{2}.$$

Let’s assume that neuron 1 satisfied this condition at time *t* = *t*′ − *dt* and fired a spike. The firing rate of neuron 1 jumps to 1 and decays thereafter, $\left(\begin{array}{c} r_{1}\\ r_{2} \end{array})\to (\begin{array}{c} \exp(-\lambda \left(t-t^{\prime }-dt\right))\\ 0 \end{array}\right)$. At time *t* = *t*′, the predicted signal jumps in the direction of *w*_1_, $\hat{s}\left(t\right)\to w_{1}\exp\left(-\lambda \left(t-t^{\prime }\right)\right)$. Since the signal is at zero, this creates prediction error, *E*(*t*′) = *a*^2^. Weight of neuron 2 points in the opposite direction than the weight of neuron 1 and neuron 2 should now spike in order to correct for the prediction error. In fact, this is likely to happen, since neuron 2 receives an excitatory PSP while neuron 1 is inhibited through the negative self-connection:

$$V_{2}\left(t\right)=ab\;\exp\left(-\lambda \left(t-t^{\prime }\right)\right)$$

$$V_{1}\left(t\right)=\frac{a^{2}}{2}-a^{2}\exp\left(-\lambda \left(t-t^{\prime }\right)\right)$$

At time *t* = *t*′, we have that *V*_2_(*t*′) = *ab* and $V_{1}\left(t^{\prime }\right)=-\frac{a^{2}}{2}$. Following the firing rule, there will be a spike of the neuron 2 if $ab>\frac{b^{2}}{2},$ which reduces to $a>\frac{b}{2}$. If for example *a* = *b*, neuron 2 will spike. In the meantime, condition for spiking in neuron 1 is $-\frac{a^{2}}{2}>\frac{a^{2}}{2}$. Neuron 1 will therefore remain silent. Spike of neuron 2 sends the predicted signal in the direction of *w*_2_.

$$\hat{s}\left(t\right)=a\;\exp\left(-\lambda \left(t-t^{\prime }\right)\right)-b\;\exp\left(-\lambda \left(t-t^{\prime \prime }\right)\right)$$

At time *t* = *t*′ + *dt* = *t*′′, predicted signal is the following: $\hat{s}\left(t^{\prime \prime }\right)=a\;\exp\left(-\lambda \left(t-t^{\prime }\right)\right)-b$. If weights have the same strength, *a* = *b*, predicted signal is now close to zero and therefore close to the desired signal. Prediction error is now *E*(*t*′′) = *a*^2^(exp(−*λ*(*t* − *t*′)) − 1)^2^. The exponential decay from one time step to the other is small and with *dt* → 0, prediction error is vanishing. Noisy spike in neuron 1 has therefore created a prediction error which was then corrected by a spike of neuron 2.

In case weights of neurons 1 and 2 are not proportional to each other, other scenarios may happen. With |*w*_1_|> > |*w*_2_|, the first noisy spike created a substantial error in the space of representation and several spikes of neuron 2 will occur in order to bring the predicted signal back to the origin. If, for example,|*w*_1_| = 10|*w*_2_|, the first noisy spike has sent the estimate far away from the origin, as far as 10 times the absolute value of the weight of the second neuron. Neglecting exponential decay due to the leak current, 10 spikes of neuron 2 are needed to bring the predicted signal back to the origin. If, on the contrary, $|w_{1}|=\frac{1}{10}\left|w_{2}\right|$, noisy spike of neuron 1 only created a small error. Now spike of neuron 2 would largely undershoot the signal and would only create a bigger prediction error. In this case, neuron 2 will not fire.

To sum up, spiking in quiescent condition can be error-correcting. A noisy spike in one neuron creates a prediction error which is then corrected by one or several error-correcting spikes of the neuron with opposite selectivity. The number of such spikes depends on the relation between the strength of neural weights and the membrane time constant, the latter controlling the strength of the leak current. Error-correcting spikes allow to maintain correct representation of the signal in spite of the noise.

#### 2.3 Inefficient spiking in the quiescent state

In an inefficient working regime, however, besides error-correcting spikes, additional spikes will be fired. What happens is that after the spurious spike of neuron 1 and the error correcting spike of neuron 2, a depolarizing PSP is sent back to neuron 1. If this PSP alone is strong enough to make the neuron 1 fire again, neuron 1 will do so, even though there is no prediction error to correct for. In such a regime, the two neurons continue to spike, correcting and again re-creating the prediction error. The time it takes to the network to “integrate” the information about a spike and react accordingly, there is already a new spurious spike that has been generated. Spiking is mechanically driven by lateral connectivity and is due to the delay in synaptic transmission, which in our case is also a delay in sharing the information about the prediction error. The requirement of a single PSP being strong enough to drive the membrane potential across threshold might seem unrealistic. However, in bigger network where neurons are allowed to synchronize, it is enough that the sum of PSPs of all neurons with opposite selectivity makes the neuron fire, which is highly plausible.

The occurrence of inefficient spiking is illustrated with a toy example. We will consider the special case where the two neurons have antisymmetric weights, **w** = (*w*_1_, *w*_2_) = (*a*, −*a*), which results in the same thresholds for the two neurons, $V_{1,2}\left(t\right)>\frac{a^{2}}{2}+\frac{\nu }{2}$. The membrane potentials of the two neurons are the following:

$$V_{1}\left(t\right)=V_{1}\left(0\right)+\int_{0}^{t^{\prime }}\left[-\lambda V_{1}\left(t\right)+a^{2}\left(o_{2}\left(t\right)-o_{1}\left(t\right)\right)\right]dt+\sigma \int_{0}^{t^{\prime }}\eta_{1}\left(t\right)dt$$

$$V_{2}\left(t\right)=V_{2}\left(0\right)+\int_{0}^{t^{\prime }}[-\lambda V_{2}\left(t\right)+a^{2}\left(o_{1}\left(t\right)-o_{2}\left(t\right)\right]dt$$

If we follow the same scenario as before, a noisy spike in neuron 1 at time *t*′ − *dt*, we get at time *t* = *t*′ an excitatory current in neuron 2 and hyperpolarization in neuron 1:

$$V_{2}\left(t\right)=\int_{0}^{t^{\prime }}\left[-\lambda V_{2}\left(t\right)+a^{2}\delta \left(t-t^{\prime }\right)\right]dt=a^{2}\exp\left(-\lambda \left(t-t^{\prime }\right)\right).$$

$$V_{1}\left(t\right)=\frac{a^{2}}{2}+\int_{0}^{t^{\prime }}\left[-\lambda V_{2}\left(t\right)-a^{2}\delta \left(t-t^{\prime }\right)\right]dt=\frac{a^{2}}{2}-a^{2}\exp\left(-\lambda \left(t-t^{\prime }\right)\right)$$

At time *t* = *t*′, the firing rule in neuron 2 is *a*^2^ > *ν* and in neuron 1 is $-a^{2}>\frac{\nu }{2}$. Since the cost can only be non-negative, neuron 1 will certainly not fire, regardless of any other parameter. Regarding the neuron 2, assume the linear cost is smaller than the square of the weight, which makes this neuron fire. At *t*′′ = *t*′ + *dt* this sends an excitatory PSP back to neuron 1 and hyperpolarizes neuron 2:

$$V_{1}\left(t\right)=\frac{a^{2}}{2}-a^{2}\exp\left(-\lambda \left(t-t^{\prime }\right)\right)+a^{2}\exp\left(-\lambda \left(t-t^{\prime \prime }\right)\right)$$

$$V_{2}\left(t\right)=a^{2}\exp\left(-\lambda \left(t-t^{\prime }\right)\right)-a^{2}\exp\left(-\lambda \left(t-t^{\prime \prime }\right)\right)$$

At time *t* = *t*′′, the firing rule for neuron 1 is $a^{2}\left(1-\exp\left(-\lambda \left(t-t^{\prime }\right)\right)\right)>\frac{\nu }{2}$. With the cost at zero, the condition for spiking in neuron 1 is again satisfied and neuron 1 will fire another spike. In an efficient working regime, however, spike at *t* = *t*′′ should not occur, since the prediction error has already been corrected with the spike of neuron 2. In order to keep spiking efficient, it is necessary to increase the linear cost. The following has to be satisfied: *ν* > 2*a*^2^ *ϵ* with *ϵ* = 1 − exp(−*λ*(*t* − *t*′)). Efficient working regime allows for the error-correcting spike, but prevents spikes that are not error-correcting, which implies the following: $2\epsilon <\frac{\nu }{a^{2}}<1$. With such relation between linear cost, weights and the strength of the leak current, there is one noisy spike followed by the error-correcting spike, after which there are no further spikes.

If linear cost is too small, i.e. *ν* < 2*a*^2^ *ϵ*, the two neurons continue to mutually excite each other. The scenario with antisymmetric weights (*w*_1_ = −*w*_2_) is particularly prone to drive long-lasting Up states. With antisymmetric weights, each spike produces the same amount of excitation (to the pair neuron) and inhibition (to itself). The amount of self-inhibition and lateral excitation to a given neuron therefore perfectly compensates, but this compensation being delayed-it occurs on subsequent time steps. With such precise but delayed balance, long Up states arise. Interestingly, noise will now be helpful to perturb this delayed balance and end an Up state. It has to be emphasized that having perfectly antisymmetric weights might not be biologically plausible, since this would require extremely precise tuning of synapses. For simulations, we used networks with multiple neurons and assumed the generic case where neural weights are randomly distributed.

### 3 Methods for statistics

All simulations are done with Matlab, Mathworks.

#### 3.1 Spike-triggered multi-unit activity

Spike-triggered multi-unit activity (S-MUA) is a measure of synchronization of single neurons with population activity. First, we compute the multi-unit activity by convolving the spike train of every neuron with an exponential kernel and sum across neurons,

$$\text{M}UA\left(t\right)=\overset{N}{\underset{i=1}{\sum }}\int_{0}^{T}u_{M}\left(t^{\prime }\right)o_{i}\left(t-t^{\prime }\right)dt^{\prime }$$ (18)

with *u*_*M*_(*t*) = exp(−*λ*_*D*_*t*), *λ*_*D*_ = 50 Hz, T is the length of the trial. The filter for convolution has short time constant, which allows to capture quick fluctuations in population firing rate. Multi-unit activity is then observed in a time window of 100 milliseconds before and after each spike, to get the non-corrected spike-triggered multi-unit activity for the neuron *i*, $SMUA_{i}^{Raw}\left(\tau^{\prime }\right)$, with *τ*′ the time lag between the spike and the multi-unit activity. Note that *τ*′ = 0 corresponds to the multi-unit activity at the time of the spike. To be able to combine results for neurons with different firing rates, we subtract from $SMUA_{i}^{Raw}\left(\tau^{\prime }\right)$ the $SMUA_{i}^{Shuffle}\left(\tau^{\prime }\right)$, where for the latter, spike train of the observed neuron and the multi-unit activity are taken from incongruent trials. *SMUA*(*τ*) is the mean across neurons of corrected spike-triggered multi-unit activity, normalized with the total number of spikes.

$$SMUA\left(\tau^{\prime }\right)=(\overset{N}{\underset{i=1}{\sum }}\int_{0}^{T}o_{i}\left(t\right)dt{)}^{-1}\frac{1}{N}\overset{N}{\underset{i=1}{\sum }}\left(SMUA_{i}^{Raw}\left(\tau^{\prime }\right)-SMUA_{i}^{Shuffle}\left(\tau^{\prime }\right)\right)$$ (19)

The peak amplitude of S-MUA is the maximal amplitude of the average S-MUA, which turned out to be at zero time lag. For estimating the S-MUA, we used 50 trials, each 200 seconds long.

#### 3.2 Duration of Inter-burst intervals and duration of Up states

Inter-burst intervals, similarly to Inter-spike intervals, measure how much time elapses between subsequent bursts/Up states. Mean Inter-burst interval is the mean across all Inter-burst intervals that occurred during a simulation trial. Distribution of Inter-burst intervals was fitted with the Gamma distribution (function “gamfit” by Matlab). The criterion for an Up state is at least 20 percent of neuron being simultaneously active. This criterion is set by hand, however, its precise value does not qualitatively change the results.

Duration of Up states measures the length of Up states. Mean duration is the mean of durations, collected during the simulation trial. Distribution of duration was not easily fitted with any unimodal distribution. For this reason, we fitted the distribution of durations with a non-parametric kernel-smoothing distribution (function “fitdist”, specification ‘Kernel’,‘epanechnikov’, Matlab). For Inter-burst intervals as well as for the duration of Up states, a simulation trial corresponding to 1000 seconds was used.

#### 3.3 Coefficient of variation 2

Coefficient of variation 2 (CV2) is the average coefficient of variation, computed from sequences of Inter-spike Intervals [14]. CV2 of neuron *i* is computed as follows:

$$CV2_{i}=2\frac{|ISI_{j+1}-ISI_{j}|}{ISI_{j+1}+ISI_{j}}$$ (20)

Mean CV2 is the average across neurons, $CV_{2}=\frac{1}{N}\sum_{i}CV2_{i}$.

## Results

It is widely recognized that neural circuits are not driven in purely feed-forward fashion. In the cortex, lateral and recurrent connectivity represent a larger portion of the synaptic inputs [4], and we can assume that they have an important role in shaping the neural code. One of the fundamental concepts on how neurons in the brain might encode behaviorally relevant variables is brought by predictive coding. Predictive coding assumes that sensory percepts are not exclusively the result of feed-forward computations, but are instead inferred from both sensory cues and predictions that are internally generated by the brain [19–21]. There is an ongoing debate in scientific community about which brain structure could implement predictive coding principles [22, 23]. Applied at a very small scale, predictive coding can take the form of a reset in single spiking neuron [24] or divisive or subtractive inhibition in neural micro-circuits [25–28]. At the level of larger populations of spiking neurons, this is equivalent to balancing excitation and inhibition as tightly as possible [29]. The principles behind predictive coding with spikes are described below. Equations and their argumentation are provided in methods. Mathematical derivations can be found elsewhere [14, 15].

### Greedy error minimization in spiking networks

**Fig 1:**
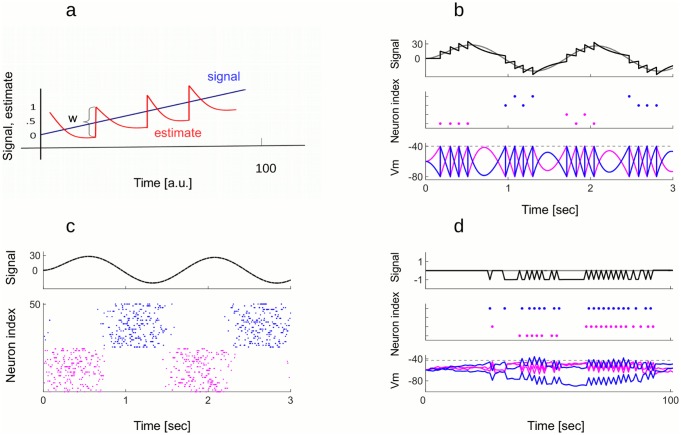
Coding signals with the model with predictive coding. A: Auto-encoder (schema). Single neuron with weight *w* = 1 represents the prediction error $\left(x\left(t\right)-\hat{x}\left(t\right)\right)$ within its membrane potential. Whenever the distance between the signal (blue) and its estimate (red) is bigger than the half of neuron’s weight, neuron spikes, which pulls the estimate towards the signal. B: Four neurons, two with weight +10 and two with weight -10, track a slow oscillating signal (gray on the upper plot). When the estimate (black on the upper plot) is too far from the signal from below, one of + neurons fires a spike (pink dots) to pull the estimate up. When the estimate is too far from the signal from above, one of—neurons fires (blue dots) to pull it down. The membrane potentials of + and - neurons are in anti-phase (lower plot). The order of firing within the + and - group is irrelevant for the read-out of spiking activity. Parameters: *ν* = 1, *μ* = 0, *σ* = 1, *λ* = 4 C: Tracking the same signal as in b) with 50 neurons, 25 with weight +0.1 and 25 with weight -0.1, gives rise to asynchronous spike trains (raster plot). Since neurons now have smaller weights and, as a network, fire more spikes, the estimation of the signal (upper plot) is much more precise. Parameters: *ν* = 1, *μ* = 0, *σ*_*thres*_ = 0.1, *λ* = 4 Hz. D: The “ping-pong” effect. 4 neurons, 2 with weight +1 and 2 with weight -1, do not receive any feed-forward drive but receive uncorrelated white noise in their membrane potentials. When one neuron fires a spike because of the noise, this activates lateral connections, provoking other spikes (middle plot). A spike of a + neuron is followed by a spike of a—neuron, which creates fast oscillation in the estimate (upper plot) as well as in the membrane potentials (lower plot). Parameters: *ν* = 1, *μ* = 0, *σ* = 0.1, *λ* = 4 Hz.

Let us first consider the simplest case, a single integrate and fire neuron with weight *w* responding to time varying input *s*(*t*). This neuron receives an input current *ws*(*t*), integrates it with a membrane time constant $\tau_{v}=\frac{1}{\lambda_{v}}$, and fires whenever the membrane potential reaches a threshold $\frac{w^{2}}{2}$. After a spike, the membrane potential is reset to $-\frac{w^{2}}{2}$. The desired signal is given by the input current, convolved with an exponential filter, the latter representing a post-synaptic potential, $x\left(t\right)=\int_{0}^{\infty }s\left(t-t^{\prime }\right)*u\left(t^{\prime }\right)dt^{\prime }$ with $u\left(t\right)=\exp\left(-\frac{t}{\tau_{v}}\right)$. The desired signal is estimated by a linear read-out of the network’s output, $\hat{x}\left(t\right)=wr\left(t\right)$. The variable *r*(*t*) is the instantaneous firing rate of the neuron, computed as a convolution of the spike train with same exponential filter as before, $r\left(t\right)=\int_{0}^{\infty }o\left(t-t^{\prime }\right)*u\left(t^{\prime }\right)dt^{\prime }$. The spike train is defined with a Dirac *δ* function *o*(*t*) = ∑_*k*_ *δ*(*t* − *t*^*k*^) with *t*^*k*^ the k-th spike of the neuron. The objective of the model is to minimize the cost function, $E\left(t\right)=(x\left(t\right)-{\hat{x}\left(t\right))}^{2}$. The neuron fires a spike whenever this minimizes the cost function, i.e. when the following condition is satisfied: *E*(*t*|*spike*) < *E*(*t*, *no* *spike*). Developing this simple rule, the membrane potential of the neuron is proportional to the coding error, $V\left(t\right)=w(x\left(t\right)-\hat{x}\left(t\right))$. The neuron performs a greedy minimization of the error: whenever the coding error exceeds a value proportional to its weight $\frac{w^{2}}{2}$ (i.e. the threshold), a new spike is fired, which decreases the coding error. As a result of the greedy minimization, the read-out of output spikes tracks the inputs as precisely as possible given *λ*_*v*_ and *w* (Fig 1a).

Exactly the same coding strategy can be performed by a population of integrate and fire neurons, working together to represent their shared signal. We assume that each time one of the neuron fires, it contributes to the estimate $\hat{x}$ according to its weight *w*_*i*_. The estimate is a leaky integration of spikes, weighted by neural weights, $\hat{x}=\sum_{i}w_{i}r_{i}$, where *r*_*i*_ is the convolution of the spike train of neuron *i*, as before. The update of the estimate after each spike is communicated to other neurons through lateral connections by −**w**^*T*^ **w**, the weight between neuron i and j being −*w*_*i*_ *w*_*j*_. As a result, the membrane potential of each neuron can be interpreted as a projection of the global coding error on its weight, e.g. $V_{i}\left(t\right)=w_{i}x\left(t\right)-\sum_{j}w_{i}w_{j}r_{j}\left(t\right)=w_{i}\left(x\left(t\right)-\hat{x}\left(t\right)\right)$. When the neuron *i* reaches a fixed threshold $\frac{w_{i}^{2}}{2}$, a new spike is fired, contributing *w*_*i*_ to the estimate which decreases the coding error $x\left(t\right)-\hat{x}\left(t\right)$ by −*w*_*i*_ in the next time step. This neuron is then reset to $-\frac{w_{i}^{2}}{2}$. Since the error remains strictly bounded by the firing thresholds, the estimate is forced to track the signal with single spike precision, as this was the case for the single neuron in Fig 1. A simple example with 4 neurons, 2 with positive weights (*w*_1,2_ = 10) and two with negative weights (*w*_3,4_ = −10), is shown on Fig 1b. When the estimate is below the signal, one of the neurons with positive weight spikes (pink dots) in order to bring the estimate up. When the estimate is above the signal, one of the neurons with negative weight spikes to pull the estimate down (blue dots). Note that any of the two neurons with the same weight is equally good for correcting the error. When the representation of the same signal is shared between more neurons, this gives rise to asynchronous spike trains (Fig 1c). A spike from any of the 25 neurons with weight +1 (pink dots) gives the same contribution to the estimate, the same being true for neurons with weight -1 (blue dots). With respect to the 4 neuron example, estimating the same signal with 50 neurons with smaller weights gives much more precise estimate of the signal (dashed black line is on the top of the gray line).

Finally, real-world stimuli may contain several variables to which neurons respond simultaneously. Neurons in the primary visual cortex, for example, are tuned to specific orientation as well as to the spatial frequency of stimuli. For the sake of generality, we therefore assume that several variables can be represented by the network simultaneously, making part of a multi-dimensional signal. Each neuron now has multiple weights, each weight corresponding to a particular input variable *s*_*j*_(*t*). Note that the signal and the estimate “live” in the same space and that the number of estimated variables is the same as the number of input variables. As before, inputs are decoded linearly from the output spike trains i.e. ${\hat{x}}_{j}\left(t\right)=\sum_{i}w_{ij}r_{i}\left(t\right)$, with *w*_*ij*_ the decoding weight of neuron *i* for the variable *s*_*j*_(*t*). ${\hat{x}}_{j}\left(t\right)$ is the estimate of *x*_*j*_(*t*), with *x*_*j*_(*t*) the convolution of the input variable *s*_*j*_(*t*), i.e. $x_{j}\left(t\right)=\int_{0}^{\infty }s_{j}\left(t-t^{\prime }\right)*u\left(t^{\prime }\right)dt^{\prime }$. The membrane potential is now the projection of the multi-dimensional coding error on neuron’s weight, i.e. $V_{i}\left(t\right)=w_{i}^{T}\left(x\left(t\right)-\hat{x}\left(t\right)\right)$ with **x**(*t*) = [*x*_1_(*t*), *x*_2_(*t*), …, *x*_*J*_(*t*)], $\hat{x}\left(t\right)=\left[{\hat{x}}_{1}\left(t\right),{\hat{x}}_{2}\left(t\right),...,{\hat{x}}_{J}\left(t\right)\right]$, with *J* the number of input variables. The threshold is now proportional to the sum of neuron’s weights across input variables: ${\text{Thres}}_{i}=\frac{\sum_{j=1}^{J}w_{ij}^{2}}{2}$.

Importantly, greedy minimization of the error with spikes does not insure that the network is efficient. It can fire many more spikes than necessary, as illustrated in Fig 1d. Consider 4 neurons with weights, pointing in opposite direction in the signal space, 2 neurons with weights +*w* and 2 neurons with weights −*w*. Neurons fire in a specific pattern: whenever neuron with positive weight fires, it excites neurons with negative weight by an amount *w*^2^, which, depending on the current state of the membrane potential, can be sufficient to bring one of those neurons to its firing threshold, $Thres=\frac{w^{2}}{2}$. If one of the neurons with negative weight fires, it in turn excites the first neuron, and so on and so forth. While the coding error still remains bounded within [−*w*, *w*], the spike count becomes absurdly large. Since neurons with opposite weights are recruited in subsequent time steps, this makes the estimate jump in +*w* and −*w* direction. We call such back and forth spiking between neurons with + and - weights the “ping-pong” effect.

Such pathological solutions can be avoided by a network minimizing not only the coding error, but the error plus cost terms penalizing high firing rates:

$$E\left(t\right)=∥x\left(t\right)-\hat{x}{\left(t\right)∥}^{2}+\mu \sum_{i}r_{i}^{2}+\nu \sum_{i}r_{i}$$ (21)

Linear cost, *ν*∑_*i*_ *r*_*i*_, and quadratic cost, $\mu \sum_{i}r_{i}^{2}$, control the relative importance of costs on spiking with respect to the accuracy of the signal estimation.

The membrane potential of the neuron *i*, derived from eq 21, is now the following:

$$V_{i}\left(t\right)=w_{i}^{T}\left(x\left(t\right)-\hat{x}\left(t\right)\right)-\mu r\left(t\right)$$ (22)

To understand how such computation could be performed by currents within the neural membrane, eq 22 is derived with respect to time. This gives the membrane equation for the neuron *i* that we use for simulations:

$${\dot{V}}_{i}\left(t\right)=-\lambda V_{i}\left(t\right)+\sum_{j}w_{ij}s_{j}\left(t\right)-\sum_{k}\phi_{ik}o_{k}\left(t\right)-\mu o_{i}\left(t\right)+\sigma \eta_{i}\left(t\right)$$ (23)

where *ϕ* = **w**^**T**^ **w** is the matrix of lateral and recurrent connections. For derivation, see methods, section 1. The noise term *ση*_*i*_(*t*) is added for biological plausibility. The noise term is a white noise with zero mean and standard deviation *σ*, uncorrelated across neurons. The standard deviation of the noise is in units of *ms*^−1^. Neuron *i* fires if the following condition is satisfied:

$$V_{i}\left(t\right)>\sum_{j=1}^{J}\frac{w_{ij}}{2}+\frac{\mu }{2}+\frac{\nu }{2}$$ (24)

The linear cost forces the network to perform the task with as few spikes as possible. The quadratic cost encourages the network to distribute spikes equally among neurons and therefore determines the distribution of firing rates. Implementing these costs corresponds to raising the firing thresholds by $\frac{\mu +\nu }{2}$ and decreasing the reset potential by −*μ*. All changes due to cost terms control the excitability of the network and it is interesting to observe that they are easily interpretable in a biological setting. Note that costs make the model more tolerant to small errors, and in particular to errors generated by network’s own dynamics. The magnitude of costs directly depends on the strength of neural weights and are therefore measured in unit of the average weight.

A noiseless network with instantaneous synapses encodes best its signals with costs at zero. If we relax these two constraints, i.e. in the presence of synaptic delays and significant neural noise, the cost terms have to be raised sufficiently high to prevent the network from constantly responding to its own internally generated errors, as explained in the next section. For analytical examples of simple networks with 2 neurons, see methods, section 2.

In the following we investigate qualitative aspects of a network with 3 input variables and 400 neurons, following eqs 23 and 24. Neural weights are drawn randomly from a standard normal distribution. The network is all-to-all connected. Set of weights is fixed and the model is not optimized for representing any particular input. Synaptic transmission has a constant delay of 1 ms, identical for all synaptic connections. The input to the network is white noise, uncorrelated across input variables, and smoothed with an exponential filter. Network parameters are listed in the Table 2 in S1 Table. This set of parameters is kept fixed, unless a particular parameter is tested for its effects.

### Consequence of synaptic delays

The predictive coding network without delays in synaptic transmission, defined by eqs 23 and 24, is optimal for tracking an arbitrary multidimensional signal. With large number of neurons, the resulting activity is typically asynchronous irregular spiking. When transmission delays are added to synapses, however, this has a dramatic effect on network activity. The network loses its asynchronous irregular working regime, since neurons massively synchronize, reflecting a population wide form of the “ping-pong”. A spike from a given neuron indeed brings not one, but several neurons with opposite coding weights to their firing thresholds. Since recurrent inhibitory weights are delayed, they cannot prevent these neurons from firing synchronously, in turn bringing several neurons with the opposite selectivity to their firing thresholds. In the most inefficient working regime with both linear and quadratic cost at zero, network quickly converges to a state where all neurons with similar selectivity fire together, in turn recruiting all neurons with opposite selectivity. However, those effects can be controlled with the cost on spiking.

**Fig 2:**
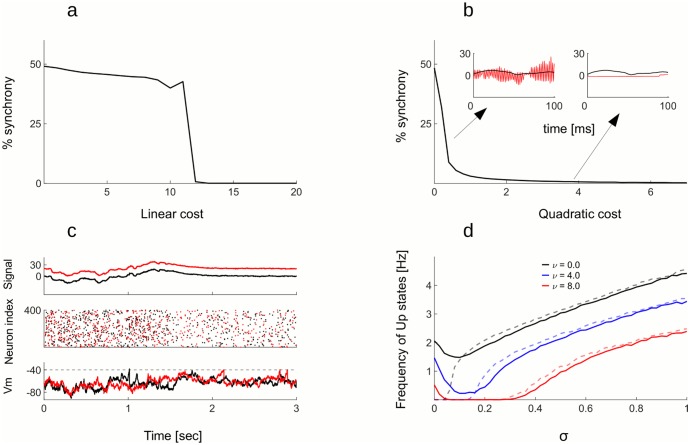
Cost on spiking controls excessive synchronization. A: Percentage of synchronously active neurons in the active state as a function of the linear cost, with no noise and with quadratic cost *μ* = 0.50 percent synchrony indicates that half of the neurons are spiking in every time step. When the linear cost is strong enough (*ν* ≈ 12), the level of synchronization suddenly drops, indicating a transition to desynchronized working regime. B: Same as in a), but now keeping the linear cost at zero and increasing the quadratic cost, the level of synchrony is now dropping progressively. Highly synchronous activity results in strong oscillations of the read-out (left inset) while for strong cost, the estimation of the signal is sluggish and imprecise (right inset). C: Network with 400 neurons in 2 simulation trials. With fixed neural weights and identical feed-forward inputs but different realizations of the noise process, there is a great variability in the realization of spike trains (middle plot, black and red dots correspond to spikes in trial 1 and 2) and in the membrane potentials of single neurons (lower plot, Vm of an example neuron in trials 1 and 2). Nevertheless, the two realizations give almost identical read-outs (upper plot, the red trace was shifted by hand). D: The frequency of Up states has non-monotonous relation to the standard deviation of the noise. For low noise levels, frequency of Up states is decreasing, it has a minimum and then starts increasing. This is true for both active (full lines) and quiescent state activity (dashed lines). Increasing the linear cost shifts the function towards lower frequency values. All other parameters are in the Table 2 in S1 Table.

Keeping the quadratic error term at zero (*μ* = 0) and increasing the linear cost term *ν* (i.e. raising firing thresholds for all neurons for the same amount), we observe a sudden transition from a state with strong synchrony and large firing rates, to synchronous irregular spiking with much lower firing rates (Fig 2a). However, even with sufficiently high thresholds this network remains highly unstable. A large enough perturbation can indeed bring it back to the regime with high firing rates.

**Fig 3:**
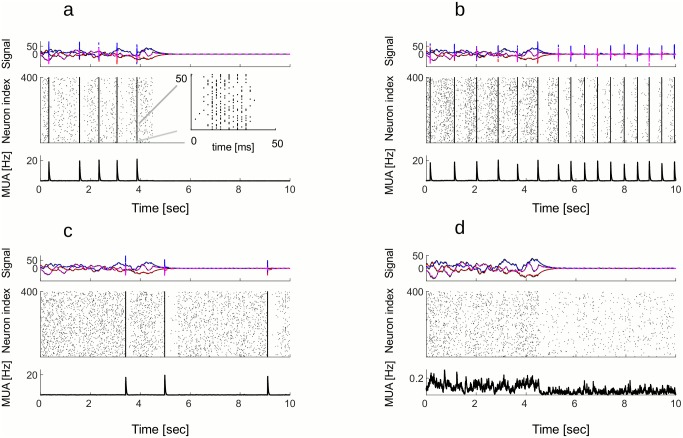
Frequency of Up states depends on the strength of the noise and the cost on spiking. A: In a network with no noise and with linear cost at zero, but with quadratic cost at *μ* = 5, short but strong Up states regularly appear in the active state. Those can be seen in the read-out (upper plot), in the spike rasters (middle plot), and in the multi-unit activity (MUA, lower plot). Without the external drive and with no noise, the network is silent in the quiescent state. INSET: A close-up into an Up state. Even though many neurons fire within an Up state, spike trains of single neurons remain irregular. B: When the noise is added to the network in A (*σ* = 0.25), Up states emerge in the quiescent state. C: When the linear cost (*ν* = 4) is added to the network in **c**, it has the effect of decreasing the frequency and the regularity of Up states. D: When the linear cost is increased even further to *ν* = 6, Up states completely disappear, only the asynchronous spiking persists. All other parameters are in the Table 2 in S1 Table.

The effect of raising the quadratic cost *μ* is markedly different. If we keep the linear cost at zero and now test the effect of quadratic cost on synchronization, the latter is decreasing progressively (Fig 2b). In addition to raising thresholds, raising the quadratic cost increases the amplitude of neural resets. As a consequence, neurons involved in an Up state become quickly hyperpolarized and stop firing, ending the “ping- pong” event. The presence of the quadratic cost prevents long Up states and stabilizes the network. However, moderate quadratic costs cannot prevent short, population wide Up states, as in Fig 3a. Note that in the working regime with moderate quadratic cost and no linear cost, Up states occur in regular intervals. There is no noise in membrane potentials, meaning that such Up states are intrinsic to the network. Naturally, the network remains silent when there is no noise and no external drive (Fig 3a, second half of the trial).

### Consequence of noise

Biological neurons operate in a noisy environment [4, 30, 31]. Realistic levels of noise have drastic consequences on the dynamics of recurrently connected networks [5, 32]. In the brain, neurons are subjected to multiple sources of noise simultaneously [30]. The synaptic noise is thought as one of the major extrinsic noise sources [4]. We first test the response of the network to the synaptic noise, simulated as a white noise, uncorrelated across units, injected in the membrane potentials of single neurons. Next, we add an intrinsic noise source, a failure in generating a spike. The failure of spike generation is simulated by imposing a probability on spiking to the neuron that has reached the threshold. If the probability of spiking is, e.g. *p*_*spike*_ = 0.3, the neuron that reaches the threshold fires a spike in 30 percent of cases. When the spike is not fired, the membrane potential remains close to the threshold and the neuron will likely fire in subsequent time steps.

When introducing the noise to the membrane potential, spontaneous activity emerges during quiescent state (Fig 3b). The activity of the network now consists of asynchronous irregular spiking as well as periods of synchronized activation of the entire network—the Up states. Raising the linear cost, Up states are getting less frequent and more irregular (Fig 3c), until they totally disappear (Fig 3d). Increasing the cost even further is not necessarily desirable since such high costs degrade coding precision. Intuitively, very high thresholds prevent all the spiking, making the network non-responsive to the noise but also to the stimuli. Fig 2d shows the frequency of Up states as a function of the noise and the linear cost, while quadratic cost is kept constant. The effect of noise on the frequency of Up states is non-monotonous. With no noise, the network easily synchronizes, resulting in regular Up states. Weak to moderate noise limits the tendency of the network to synchronize and may improve coding accuracy, a form of stochastic resonance. However, if the noise is increased further, the frequency of Up states again increases, since Up states are now also triggered by the noise. Higher costs result in less Up states at all levels of noise. Interestingly, for high costs and moderate levels of noise, the Up states disappear entirely.

**Fig 4:**
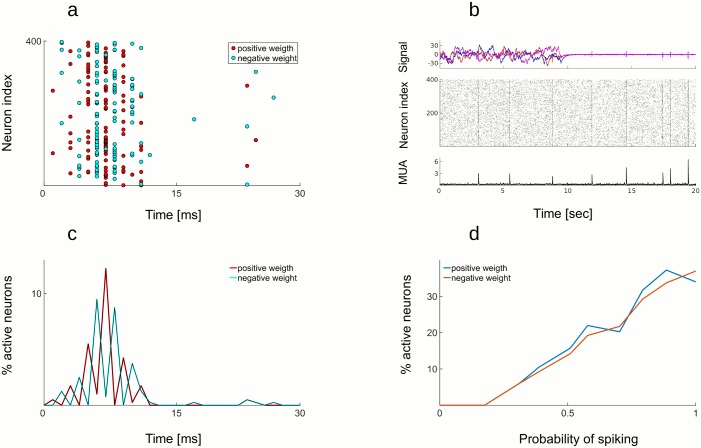
An Up state arises because of synchronous spiking of neurons with same selectivity. A: Close-up in an Up state. Red dots are spikes of neurons with positive weights and cyan dots are spikes of neurons with negative weights. During an Up state, neurons with same selectivity fire synchronously. The sub-populations of neurons with positive and negative weights fire in alternation. B: Same as plots in Fig 3, but with a secondary noise source, the synaptic failure. By including synaptic failure, we obtain more realistic Up states. C: Percentage of active neurons during the Up state in A. Red trace is for neurons with positive weights and cyan trace is for neurons with negative weights. Only spikes corresponding to a single dimension of the stimulus are decoded. D: Percentage of neurons that activate simultaneously at the peak of an Up state as a function of probability of spiking. Percentage of active neurons increases with probability of spiking. Parameters: *ν* = 3, *μ* = 3, *p*_*spike*_ = 0.3. All other parameters are in the Table 2 in S1 Table.

Within an Up state, a large fraction of neurons is recruited (in Fig 3a, around 60 percent). However, the order in which the neurons are recruited is random (Fig 3a, inset). Many neurons fire within the same synaptic delay, but most neurons fire no more than two or three times within an Up state. The Up states as shown on the Fig 3 are transient epochs of very strong synchronization, which is a consequence of dense connectivity. The strength of synchronization can be modulated by introducing the secondary source of noise, the failure of spike generation. By keeping the noise in the membrane potentials and including the failure in spike generation in the model, we obtain activity that more closely resembles a class of working regimes in the brain (Fig 4b). A close-up into an Up state shows the transient period of synchronous spiking (Fig 4a). Separating spikes from neurons with positive and negative weights (red and cyan dots respectively), the Up state consists of alternative spiking of the two sub-populations on consecutive time steps (Fig 4a and 4c). Percentage of neurons that activate at the peak of an Up state depends on the probability of spiking (Fig 4d). Neurons with similar or opposite selectivity, i.e. with their weight vectors pointing in the same or opposite directions in signal space, tend to be recruited within the same Up state while neurons with independent selectivity, i.e. with orthogonal weight vectors *w*_*i*_*w*_*j*_ = 0, tend to be recruited in different Up states. The reason can be intuitively understood as an extension of the “ping-pong” dynamics to the 3 dimensional signal space. Note that the secondary source of noise has the effect of decreasing synchronization within an Up state, but it does not trigger Up states on its own. Also, it does not have an effect on coding properties within an Up state. For this reason we do not include it in the further analysis of the all-to-all connected network.

**Fig 5:**
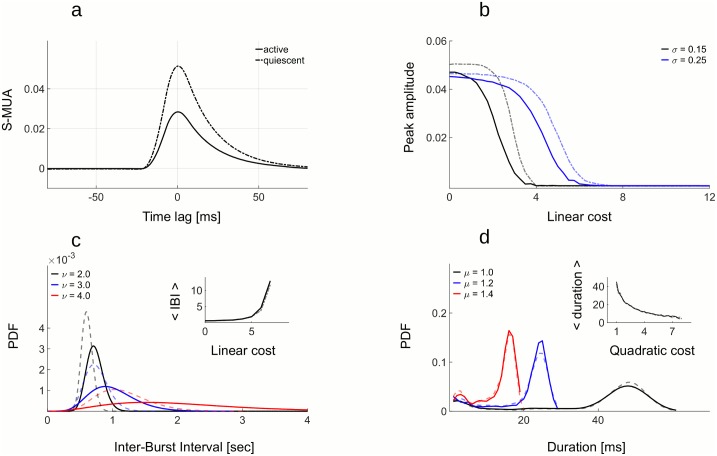
Statistics of Up states in active and quiescent state. A: Mean spike-triggered multi-unit activity (S-MUA) in active (full line) and quiescent state (dashed line) for the network with noise (*σ* = 0.25) and moderate costs (*μ* = 5, *ν* = 5.75). Temporal locking of single neuron activity to the population activity is stronger in the quiescent (dashed line) compared to the active state (full line). B: Peak amplitude of S-MUA decreases with increasing linear cost in both active state (full line) and quiescent state (dashed line). With stronger noise, time-locking of single neurons with the population activity persists for a wider range of linear costs (compare blue and black lines). For strong enough costs, there is no more time-locking. C: The Inter-burst Intervals (time intervals between succesive Up states, shorter IBI) are modulated by the linear cost in their mean and their distribution. Mean IBI increases with the linear cost (inset). For small linear costs, distribution of Up states is narrow, indicating that Up states occur frequently and in regular intervals (black line, full for active and dashed for quiescent state). For stronger costs, distribution of Inter-burst Intervals is getting wider and is moving to the right, indicating less frequent Up states with irregular timing (blue and red lines). D: The mean and the distribution of Duration of Up states is controlled by the quadratic cost. With increasing quadratic cost, mean Duration of Up states is decreasing (inset) and the distribution is getting wider. All other parameters are in the Table 2 in S1 Table.

To evaluate the amount of synchronization of single neurons with the population activity, we measure the mean spike-triggered multi-unit activity (S-MUA, Fig 5a and 5b). S-MUA was measured for entire simulated trials, including both synchronous Up states and asynchronous activity. Synchrony is stronger in quiescent state compared to the active state (Fig 5a). The peak amplitude of S-MUA, if it exists, happens to be at the zero time lag, meaning that neurons are most likely to spike when the rest of the network is also active. The synchronization of single neurons with the rest of the network is strongly modulated by the linear cost (Fig 5b). With increasing linear cost, synchronization is getting weaker and for sufficiently high cost, neurons completely desynchronize. This is true for both active and quiescent states. The setting of costs also determines the temporal regularity of Up states. Up states are frequent and regular, with narrowly distributed inter-burst-intervals, for low levels of linear cost, but they become increasingly rare and irregular at higher linear costs (Fig 5c). Up states are more regular and more frequent in the quiescent than in the active state (Fig 5c, compare dashed versus full lines). Quadratic cost determines the duration of Up states, the latter getting shorter with increasing quadratic cost (Fig 5d).

### Up-states and efficient coding

As seen in Fig 2a and 2b, the network with synaptic delays has to be controlled by costs on spiking in order to prevent excessive synchronization. In fact, excessive synchronization also deteriorates coding precision of the network. We call “efficient” a network that is both accurate in representing the input signals as well as parsimonious with spikes. In this section we quantify the effect of cost parameters on network efficiency. We define an efficiency measure, which takes into account the coding precision and the average number of spikes that are “spent” for achieving such precision. We define the mean coding error as the average prediction error over the simulation trial:

$$\left\langle error\right\rangle =\frac{1}{{\left\langle ∥w∥\right\rangle }_{i}}\frac{1}{TJ}\int_{0}^{T}\sum_{j=1}^{J}∥x_{j}\left(t\right)-{\hat{x}}_{j}\left(t\right)∥dt$$ (25)

where *T* is the length of the trial and *J* is the number of input variables. The mean error is rescaled with the mean norm of weights, where the mean is taken across neurons. The effect of a spike on the prediction error depends on the weight of the active neuron. To make the mean error independent on a particular choice of the scale of **w**, the mean error is normalized with an average norm of **w**. With the average across neurons we implicitly assume that all neurons fire with same firing rates.

To measure the mean cost, we count the mean number of spikes in a simulation trial:

$$\left\langle cost\right\rangle =\frac{1}{T}\int_{0}^{T}\sum_{i=1}^{N}o_{i}\left(t\right)dt$$ (26)

The 〈*error*〉 is the temporal average of the distance between the signal and its estimate and the 〈*cost*〉 is the average population firing rate. The weighted sum of the two is the *Total* *error*

Totalerror=α⟨error⟩+β⟨cost⟩ (27)

**Fig 6:**
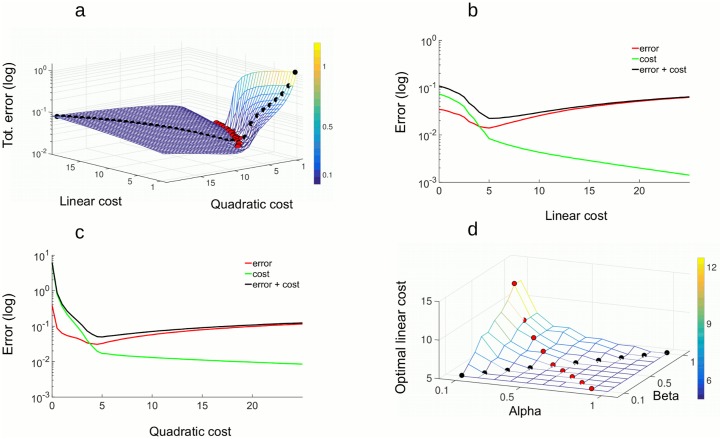
Efficiency of the model. A: *Total* *error* with *α* = *β* = 1, evaluated jointly for a range of linear and quadratic cost parameters. Black dots are the *ν* = *μ* identity line. Red dots are 20 points where the *Total* *error* is the smallest. The z-axis uses the scale of the natural logarithm. B: *Total* *error* as a function of the linear cost, for quadratic cost fixed at *μ* = 5. The y-axis uses the scale of the natural logarithm. C: *Total* *error* as a function of the quadratic cost, for linear cost fixed at *μ* = 5. D: Optimal linear cost as a function of weighs *α* and *β* from *Total* *error* = *α*〈*error*〉 + *β*〈*cost*〉. Red points indicate the *ν* = *μ* line and red points indicate the subspace along which *α* + *β* = 1. All parameters are in the Table 2 in S1 Table.

The encoding of signals is maximally efficient when the *Total* *error* is minimized. The *Total* *error* can be evaluated as a function of any network parameter. Here we test it with respect to linear and quadratic cost parameters. The *Total* *error* is measured during the active state, with smoothed white noise as the input and with a single noise source, the white noise in membrane potentials. Minimizing jointly the linear and the quadratic cost parameter, the *Total* *error* is more sensitive to the quadratic than to the linear cost (Fig 6a), hence the scale with natural logarithm. There is a region where the *Total* *error* is best minimized (Fig 6a, red dots). This region is approximately perpendicular to the identity line *ν* = *μ* (black dots). Overall, this implies that there are multiple “good solutions” for the fine tuning of neural thresholds and spike-triggered adaptation.

**Fig 7:**
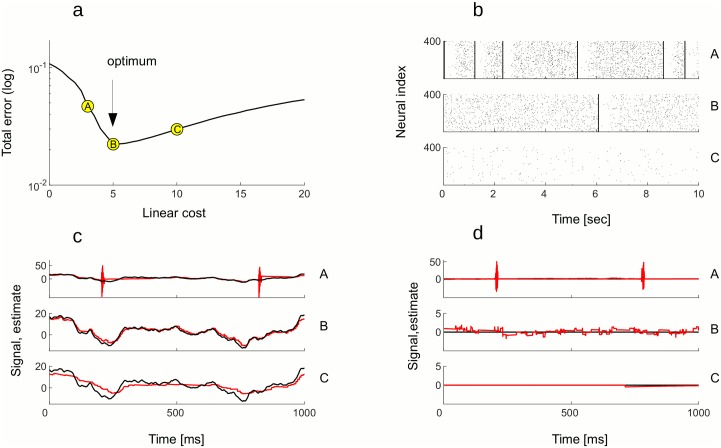
Effects of efficiency on the network dynamics and the read-out. A: Moving along the *Total* *error* function, point B indicates the maximal efficiency and points A and C indicate two qualitatively different suboptimal working regimes. B: Raster plot of activity in the quiescent state (no stimulus), corresponding to network parameters in A, B and C. In the optimal working regime (middle plot), network shows desynchronized spiking due to the noise and occasional, albeit very rare, moments of synchronous bursts of activity. In suboptimal working regime corresponding to A, the network is easily excitable (upper plot) and the frequency of spontaneous bursts increases. In the working regime corresponding to C, network is less responsive (lower plot), hence little or no spikes will be fired in the absence of the stimulus. C: Population read-out in the active state. In the optimal working regime (middle plot), population read-out (red trace) gives the best estimate of the desired signal (black trace). In the suboptimal regime with easily excitable network, the estimate of the signal is occasionally perturbed by strong oscillations, the network synchronizing and “over-representing” the signal with too many spikes (upper plot). When the network is less responsive, the estimation of the signal is sloppy and imprecise (lower plot). D: Population read-out in the quiescent state. In the optimal regime (middle plot), read-out of population activity oscillates around zero, its desired/true value. In the regime with easily excitable network (upper plot), occasional bursts provoke strong oscillations of the read-out. When the excitability is suboptimal but low (lower plot), the population read-out is constantly at zero. All other parameters are in the Table 2 in S1 Table.

Fixing the quadratic cost and testing the efficiency of the network as a function of the linear cost, the *Total* *error* has a minimum, indicating the most efficient working regime for this particular setting of the quadratic cost (Fig 6b). We observe that the 〈*error*〉 function alone has a minimum, which approximately coincides with the minimum of the *Total* *error*. The minimum of the *Totalerror* also coincides with a point where the 〈*cost*〉 changes behavior from steeply dropping to slowly decreasing. This is consistent with the transition from working regimes with frequent Up states to regimes with rare or no Up states (Fig 7b). Increasing costs beyond the minimum, however, results in an increase of the coding error (Fig 6b, red trace after the minimum). Even if these higher costs completely eliminate spontaneous Up states (Fig 7c and 7d), the benefit in term of further decreasing spike counts is moderate and does not compensate for the growing coding errors. In particular, a network with high costs, corresponding to high firing thresholds and large resets, will fail to respond to weak stimuli, to track fast varying stimuli, or correct errors induced by noise. Similar functions result from fixing the linear cost and measuring the 〈*error*〉, and the 〈*cost*〉 as a function of the quadratic cost parameter (Fig 6c).

In general, the *Total* *error* is defined as a weighted sum of the 〈*cost*〉 and the 〈*error*〉. The minimum of the *Total* *error* stays constant for equal weighting of the 〈*cost*〉 and the 〈*error*〉 (*α* = *β*, black dots on the Fig 6b) and when *α* > *β*, but increases exponentially along the *α* + *β* = 1 line (red dots), namely after the point where the *β* parameter is dominant (*β* > *α*). In such a regime, the network would be less responsive to stimuli and would remain silent in the quiescent condition. We argue that weighting equally the 〈*error*〉 and the 〈*cost*〉 is the most relevant case to consider, since in this case a single spike contributes a unit to the error and a unit to the cost. While the contribution to the cost can only be positive, a spike can either decrease the error (efficient spike) or increase it (inefficient spike, see Methods, section 2). For costs bigger then the optimal, a vast majority of spikes is efficient while for costs smaller than the optimum, many spikes are not. The optimal working regime is where a maximal number of efficient spikes occur.

**Fig 8:**
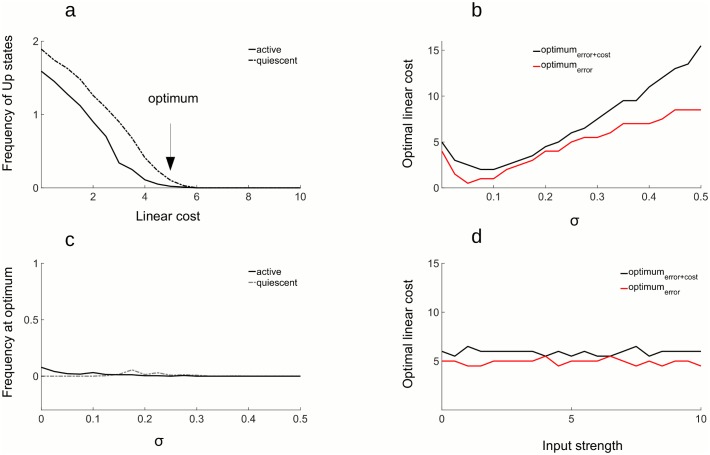
Characteristics of the optimal network. A: Frequency of the Up states as a function of the linear cost. Frequency of Up states decreases with increasing linear cost. At the optimal cost, indicated by an arrow, the frequency of Up states is close to zero, indicating that in the optimally efficient network, Up states are at the point of vanishing. **b**) Optimal linear cost in function of the strength of the noise. Optimal linear cost is modulated by the strength of the noise (i.e. the *σ* of the noise process) in a non-monotonous fashion. For small noise levels, the optimal cost is decreasing, reaches a minimum and increases thereafter. This is true for the optimal cost estimated from the *Total* *error* (black trace), as well as for the optimal cost estimated from the coding error only (red trace). **c**) Frequency of Up states for the networks with optimal costs in function of the strength of the noise. At the optimum, frequency of Up states is always close to zero, irrespective of the level of the noise. This is true for both active (full line) and quiescent state activity (dashed line). **d**) Optimal linear cost does not depend on the strength of the input. For a reasonable range of input strengths, optimal cost stays constant. All other parameters are in the Table 2 in S1 Table.

As it follows from its definition, the efficiency has effects on both the coding capacity of the network as well as on its dynamics (Fig 7). The maximal efficiency (Fig 7a, point B at the minimum of the *Total* *error*) corresponds to the best coding capacity in the active state (Fig 7c) and to the regime where the Up states are at the point of vanishing in the quiescent state (Fig 8a). This result is robust to changes in network size, noise level (Fig 8b and 8c), and signal strength (Fig 8d). In all parameters investigated, optimal efficiency occurs near the point when Up states disappear, but not beyond (Fig 8c). Thus, the most efficient network is in a as high-gain regime as possible, with spontaneous Up states still present albeit rare and irregular.

Important to consider is that the optimal cost depends on the strength of the noise, this dependence being nonlinear and non-monotonic (Fig 8b). Interestingly, for the low level of noise the optimal cost is decreasing with the strength of the noise, indicating that some amount of noise in fact helps the network to be more efficient. This is the regime where the noise is too weak to trigger spiking. For stronger noise levels, when the noise is strong enough to provoke spiking, optimal cost is increased to prevent triggering of Up states. Since the strength of the noise and the linear cost have a nonlinear relation, it is not possible to simply rescale one with the other. For a given level of noise, it is always possible to find an optimal cost for spiking. Testing the network for any such *ν*|*σ* pair, the Up states are at the point of vanishing (Fig 8c).

Since spontaneous activity and Up states have an important effect on the population activity, why do they not degrade sensory coding more severely (e.g., see Fig 6b, the 〈*error*〉〉 before the optimum is decreasing, but not as steeply as the 〈*cost*〉)? Intuitively, this is because the error-correcting mechanism is still functional and the network constantly corrects its own “mistakes”. Consider a toy example of two neurons, one with weight +*w* and one with weight −*w*, that do not receive any feed-forward input but receive the noise in their membrane potentials. Neurons + and − are interconnected with an excitatory connection, −(*w*_1_ *w*_2_) = −(*w*(−*w*)) = *w*^2^. The desired signal is at zero at all times. If, by chance, the noise builds up in the membrane potential of one neuron, this neuron fires. With a noisy spike, the estimated signal is sent away from zero, in the direction of the weight of the spiking neuron. This creates a prediction error. When the first neuron spikes, it also sends an excitatory PSP to the neuron with opposite selectivity, making it more likely to spike in the next time step. The spike of the second neuron is in fact efficient, since it brings the prediction error back to the origin. In the efficient working regime, there is no more spiking thereafter. However, the second spike in turn depolarizes the first neuron. In the inefficient regime, where neural thresholds are not adjusted, the first neuron fires again, which re-creates the prediction error. Such “back and forth” spiking between the two neurons with opposite selectivity alternates efficient and inefficient spikes. For mathematical illustration, see Methods, section 2.

**Fig 9:**
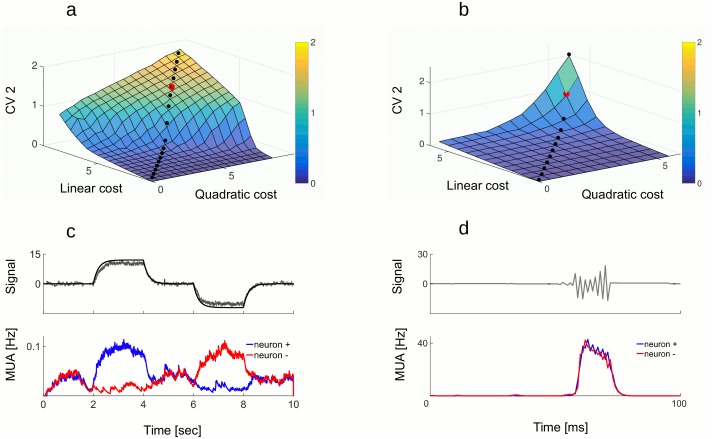
Signatures of efficient coding in active and quiescent states. A: Average Coefficient of variation for a sequence of Inter-spike Intervals (CV 2) in active state is modulated by the linear and the quadratic cost. Black dots indicate the line along which the costs constants are equal, i.e. *μ* = *ν* and the red dot indicates optimal costs. For optimal costs, CV 2 is slightly below 1, meaning that spiking is close to Poisson, albeit more regular. B: Same in the quiescent state. CV 2 for optimal costs is close to 1, indicating a Poisson process. C: Decoding stimulus-driven activity of + and − subpopulations. In the active state, activation of + and − neurons depends on the behavior of the signal. When the signal (black line on the upper plot) increases in the + direction, the subpopulation of + neurons (blue trace on the lower plot) increases their firing rates. When, on the contrary, signal decreases in the − direction, subpopulation of − neurons (red trace) strongly activates. D: Decoding an Up state. In the absence of the stimulus, neurons either fire isolated, noise-induced spikes or engage in highly synchronized spiking during an Up state. Synchronized spiking has a particular and salient decoding pattern. Since + and − neurons fire in quick alternation, they create a fast oscillation of the read-out around zero (upper plot). Firing rates of both + and − populations rise and decay together (lower plot). All other parameters are in the Table 2 in S1 Table.

In a more realistic setting, consider the response of two sub-populations of neurons to one dimensional signal: + neurons (with positive weights) respond to an increase in signal above the baseline, while − neurons (with negative weights) respond to a decrease in signal below the baseline (Fig 9c). Something completely different happens during an Up state. In that case, both + and − neurons are recruited equally within the same Up state (Fig 9d). They largely cancel each other’s effect such that, on average, no stimulus is encoded, despite the sharp increase in population rate. Superficially, we observe a sudden increase in firing in both cases, but the two activities have different “meaning” when spiking is decoded. While stimulus-locked transient response recruits only neurons of similar selectivity, in order to respond to a sudden change in the signal, + and − neurons are recruited in alternation during an Up state, resulting in an estimate that is oscillating around zero. This particular decoding pattern of Up states is a prediction of the model that could be tested experimentally, by estimating the decoding weights of neurons during the active state and decoding spikes during quiescent state. A less salient, although interesting characteristics of the optimal network is to have the CV2 (see methods, section 3.3) slightly below 1. For a Poisson process, CV2 is 1. Slightly more regular spiking than the Poisson process is due to the presence of occasional Up states, but otherwise indicates the proximity of an asynchronous spiking regime.

While useful to illustrate the mechanism and the impact of Up states in a noisy balanced network performing efficient coding, many aspects of this model are not generally applicable. In particular, the model we studied so far has all-to-all connections and the model might apply to densely connected microcircuits. In the next section, we build a spatially organized layer of neurons with local connectivity, and test whether conclusions about network efficiency generalize to such cases.

### Traveling waves in spatially organized networks with local connectivity

We now construct a topographic network with input signals corresponding to one dimensional “image”, composed of a number of spatially arranged pixels. We assume the input is a circular variable with Gaussian statistics:

$$s_{j}\left(t\right)=A\exp\left(B\left(\cos\left(\theta_{j}-c\left(t\right)\right)-1\right)\right)$$ (28)

with *θ*_*i*_ ∈ [0, 2*π*] and elements equally spaced, *θ*_*i*+1_ − *θ*_*i*_ = *const*. The variable *c*(*t*) is smoothed white noise, $c\left(t\right)=\int_{0}^{\infty }\eta \left(t-s\right)u_{2}\left(s\right)ds$, *u*_2_(*t*) = exp(−*λ*_*input*_ *t*). Parameters A, B and *λ*_*input*_ as well as other parameters of the spatially organized network can be find in the Table 3 in S1 Table.

Neurons encode input variables with local weights or receptive fields, representing a blob-shaped increase (ON neurons) or decrease (OFF neurons) in e.g. the luminance of the stimulus. There are 200 ON neurons:

$$w_{ij}=C\exp\left(D\cos\left(\frac{2\pi (\theta_{j}-\theta_{i})}{N}\right)-1\right),$$ (29)

and 200 OFF neurons:

$$w_{ij}=-C\exp\left(D\cos\left(\frac{2\pi (\theta_{j}-\theta_{i})}{N}\right)-1\right)$$ (30)

Note that ON and OFF neurons with the same peak position (either positive of negative) are considered to be at the same physical position on the layer. Neurons therefore share the same spatial organization as the input and respond to the input only if the latter is inside their receptive field. Because only nearby neurons have non-orthogonal decoding weights, connections in the network are local. Similarly to what we had before, connections between neurons of the same polarity are inhibitory, while connections between neurons of opposite polarity are excitatory. There is a 2 ms synaptic delay.

**Fig 10:**
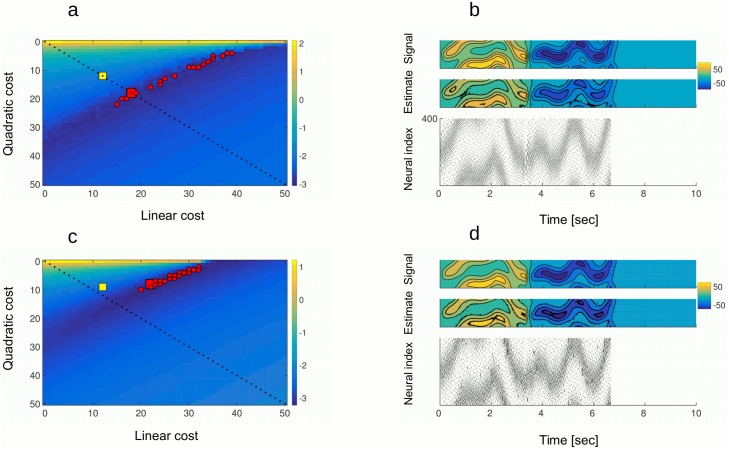
Spatially organized network. A: The natural logarithm of the *Total* *error* as a function of linear and quadratic cost parameters. Black dots are the *ν* = *μ* identity line and red dots are 20 points that best minimize the *Total* *error* with respect to the linear and quadratic cost parameters. Yellow square marks a suboptimal regime with costs smaller than optimal. B: Activity of the network, with cost parameters corresponding to one of the optimal settings for cost parameters (red square in A. The signal (upper plot) is accurately represented by the estimate (middle plot). At the beginning of the trial, the network tracks a signal with positive sign. Next, it tracks a signal with negative sign. The trial ends with quiescent period. On the raster plot, ON and OFF neurons are interleaved. C: Same as in A, for the network with an additional noise source, the failure in spike generation. D: Activity of the network, with cost parameters corresponding to the red square in C where costs are suboptimal. The representation of the signal is still accurate, with occasional short Up states in the active state. No Up states are observed in the quiescent condition. Parameter for A and B: *p*_*spike*_ = 1, parameter for C and D: *p*_*spike*_ = 0.3. All other parameters are in the Table 3 in S1 Table.

**Fig 11:**
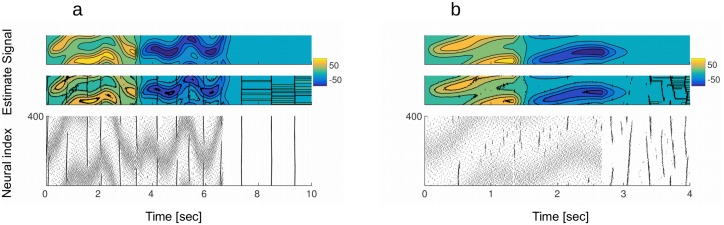
The activity of the spatially organized network in suboptimal working regimes. A: The signal (upper plot), the estimate of the signal (midle plot) and the spike raster (lower plot) for the network with local connectivity and the noise in the membrane potentials. In suboptimal working regime with linear and quadratic cost parameters smaller than optimal, (yellow square in Fig 10a, (*ν*, *μ*) = (12, 12)), Up states emerge in active and quiescent state. The signal can nevertheless be represented. Parameter: *p*_*spike*_ = 1. B: Same as in A, with additional noise source, the failure in spike generation. Cost parameters are smaller than optimal and correspond to the yellow square in Fig 10c with (*ν*, *μ*) = (12, 10). Parameter: *p*_*spike*_ = 0.3. Other parameters are in the Table 3 in S1 Table.

In order to find an efficient working regime, we test the network with the efficiency measure. Similarly to the all-to-all connected model, the *Total* *error* function in the spatially organized network allows to estimate optimal cost parameters. The dependency of the *Total* *error* on the linear and quadratic cost constants behaves similarly to the one with the all-to-all connected model. As before, the *Total* *error* is more sensitive to the quadratic than to the linear cost, hence the plot of the natural logarithm of the error, *log*(*Total* *error*) (Fig 10a and 10c). Notice that in general the logarithmic operation does not change the minimum of the function. There is a region where the *Total* *error* is jointly minimized for the linear and the quadratic cost parameters (red dots), roughly perpendicular to the *μ* = *nu* identity line (black dots). The activity corresponding to one of the best settings of costs, (Fig 10a, red square), results in an accurate representation of the stimulus (Fig 10b, upper and middle plots). There are no Up states in this working regime (Fig 10b, lower plot). In suboptimal regime with costs at (*ν*, *μ*) = (12, 12), marked by yellow square on Fig 10a), Up states appear in both active and quiescent state (Fig 11a).

**Fig 12:**
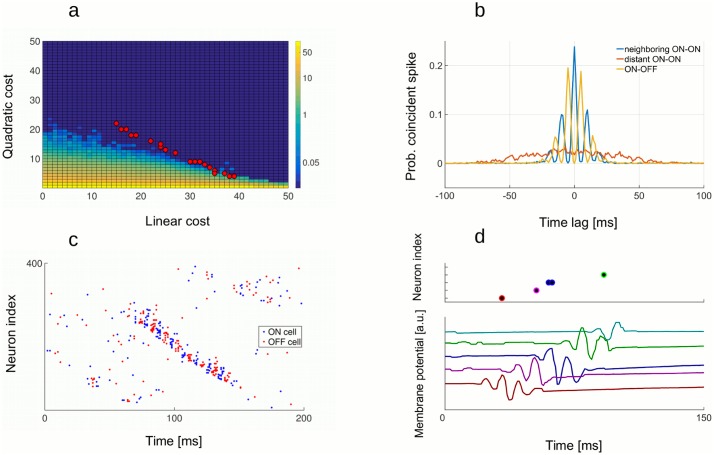
Traveling waves. A: Percentage of time in the Up state as a function of cost parameters for the network with single noise source. For very low costs, the network is permanently in the Up state. For high costs, Up states never occur. The transition between the region with Up states and the region without those approximately coincides with points of minimal error (red dots). B: Cross-correlograms of quiescent state spiking between neighboring cells with same selectivity (blue), opposite selectivity (red) and between distant cells (red). Selectivity relation between a pair of cells determines very distinct cross-correlogram profiles. Neighbors with same selectivity fire in phase while neighbors with opposite selectivity fire in anti-phase. Distant cells do not phase-lock their spike timing. C: An Up state is traveling through the network by engaging local connections between cells of neighboring selectivity. Clusters of ON cells (blue dots) and OFF cells (red dots) are activated in alternation. D: Close-up in an Up state shows a perturbation in the membrane potentials of single neurons that is transmitted locally to neighboring neurons (lower plot). While the perturbation of membrane potentials propagate to all local cells, spiking pattern from one cell to another is irregular or absent. All parameters are in the Table 3 in S1 Table.

Finally, we also test the efficiency of the network with an additional noise source, the failure of spike generation. Compared to the model with a single noise source, cost parameters that minimize best the *Total* *error* are now confined to lower quadratic costs and higher linear costs (Fig 10c, red dots). Activity corresponding to a set of optimal costs (*μ*, *ν*) = (9, 21) (Fig 10c, red square) is again characterized by accurate representation of the signal and the absence of Up states (Fig 10d). In the suboptimal working regime with (*μ*, *ν*) = (10, 12), corresponding to the yellow square on Fig 10c, Up states appear in both active and quiescent condition (Fig 11b). Since Up states recruit equally ON and OFF cells, they have a minimal impact on the representation of the signal (Fig 11). In particular, a silent signal is represented during quiescence, despite the abundance of spiking. Localized spontaneous bursts appear for a range of costs smaller than the optima (Fig 12a). This is true for the model with single noise source as well as for the model with two noise sources. The only qualitative effect of the secondary noise source is less strong synchronization during an Up state, which results in Up states that more closely resemble spontaneous bursts of activity that are observed in biological networks. Notice that the effect of the failure in spike generation is therefore the same as in the all-to-all connected network.

In contrast to the all-to-all connected model, Up states in the model with local connectivity are traveling through the network. Synchronization can now recruit only cells in a local portion of the layer, i.e. cells with similar receptive fields. Despite this fact, Up states often engage a big portion of the neural layer (Fig 12c). We can investigate further the mechanism behind these waves of activity by looking intra-cellularly at the membrane potentials. Bursts of activity are caused by local perturbation of membrane potentials (Fig 12d) and progressively propagate to cells of nearby selectivity (Fig 12c). During a burst, spikes ride on the top of brief periods of fast oscillations, clearly visible in the membrane potentials. During an Up state, the temporal envelope of these oscillation travels locally, reflecting the propagation of the initial perturbation through lateral connections of nearby neurons. Oscillations in membrane potentials of ON and OFF cells are in anti phase (Fig 12b). Even if these oscillations propagate to all local cells, only a proportion of cells actually fire. Moreover, a cell might not fire in each oscillation cycle (on Fig 12d, red, magenta and green cells fired one spike while blue cell fired two spikes and green cell fired none). As a result, single cell’s spike trains appear irregular both during and outside of the Up state, while the underlying fast perturbation of membrane potential is present in all cells and travels though the network. In comparison with the all-to-all connected network, Up states in the model with local connectivity therefore rely on same computational mechanism, but in addition show traveling property because of the spatial extension of the model. As observed beforehand, duration of waves is highly sensitive to the cost on spiking. Costs larger than optima lead to the absence of spontaneous waves during quiescence, and eventually to the complete absence of spontaneous activity. In contrast, costs smaller than optima result in an increase of the frequency and the duration of the waves, until they occur continuously during both stimulus presentation and quiescence (Fig 12a). Traveling waves of activity have recently been reported in V1 and V2 areas of the monkey in both active and quiescent states [33, 34].

## Discussion

Predictive coding aims at representing an arbitrary time-varying signal as accurately as possible and with minimum number of spikes. The model performs a population code, where the representation of the signal arises from joint activity of all the neurons in the network. When subjected to perturbations, such a network will correct the erroneous representation of input variables with additional spiking. In the quiescent state, spiking activity follows the same computational principles as in the active state and the network now represents a silent (zero) signal. Spiking in quiescent periods is triggered by the noise, which creates a prediction error. The prediction error activates the self-correcting mechanism, which, depending on the cost on spiking, corrects the initial error more or less efficiently. The self-correcting mechanism reflects the computation, performed by the network. We propose the self-correcting mechanism as a candidate mechanism to account for spontaneous activity in recurrently connected networks.

While quiescent state activity can be mechanistically accounted for by activation of lateral connections in recurrently connected circuits (see [8, 35] for in vitro, [7] for in vivo and [9] in computo), it is less clear what is the “explanation” of spontaneous activity in terms of computation. The present work suggests that even an abundant spiking in the quiescent state might not be arbitrary noise, but is instead a consequence of precise but inefficient computation. An Up state in the quiescent condition can be understood as an attempt to maintain correct representation of a silent signal in the presence of noise. Decoding the internal estimate of the signal in the latter situation shows that the internal representation during an Up state oscillates around zero and is only minimally interfering with processing of the stimuli, despite the apparent variability in the response. Decoding quiescent state activity could in principle result in an arbitrary signal. If, in contrast, spikes are aimed at minimizing the coding error, the network chooses a particular solution which is an oscillation around zero, the real value of the stimulus and the desired value of the computation. This particular solution allows avoiding an arbitrary erroneous representation and enforces the network to remain close to the correct representation at all times.

So far, a variety of hypotheses have been raised to explain the functional role of spontaneous activity. Spontaneous activity has been related to the replay of the sensory experience [36], reorganization of synaptic weights in the network [37], processing of the past experience [38], memorization of sensory events [39, 40], bottom-up thalamic control [41] and top-down modulation [42]. In contrast to cited studies, the present work accounts for spontaneous activity by considering it as an extension of a computation that the network might be performing with its internal signals. It can be argued that the predictions of our model contrast with functional accounts of spontaneous activity as a replay of sensory signals. During a replay, quiescent network is re-activated, showing the same or similar pattern of activity as during the presentation of the stimulus. Importantly, it also represents one of its past signals. The read-out of the neural activity should extract that signal as if it was an actual sensory response. On the contrary, a network with predictive coding ensures that the internal signal in quiescent state stays around zero, even though the noise triggers activity. Decoding these activities should therefore not find any representation of a past signal during quiescence and during Up states.

Qualitatively different responses that change with the behavioral state of the animal have been reported by several studies using electrophysiology [1, 7, 42, 43]. When the animal is inactive, neural activity is characterized by low frequency fluctuations of the membrane potential and by bursts of spontaneous spiking, followed by periods of silence. While actively behaving, on the other hand, membrane potential fluctuations are of high frequency and the network is in a desynchronized state. In our model, we have observed similar phenomena. From active to quiescent state, the level of synchronization of single neurons with the network increases. This happens automatically when the external input is set to zero and does not require any change in model parameters. By changing the costs on spiking, which have simple biological explanations as changing the excitability of the network, the model shows a continuum of states, similarly to what has been observed in aforementioned studies. Relating network dynamics to the network function, our work suggests that the two are closely inter-dependent. Predictive coding models give a simple account on observed qualitative changes of the network activity for the continuity of states, from alert active state to states with low level of alertness. In vivo recordings in active and quiescent prefrontal cortex have indeed demonstrated that neural responsiveness is modulated on-line, presumably in a behaviorally relevant manner [13].

From models with predictive coding it follows that costs on spiking determine the working regime of the network. Change in costs can be understood as the modulation of neural activity by the behavioral state in biological networks. When the animal is actively behaving, the cost on spiking would presumably be optimal because accurate representation of a signal is a priority, resulting in high responsiveness and giving rise to quickest and most efficient corrections of the prediction error. Conversely, when the animal is inactive, the cost on spiking might be suboptimal, resulting in more frequent Up states or else in sluggish representation of the input signals with little spiking.

Within the predictive coding framework, we interpret the quadratic cost as the spike-triggered adaptation and the linear cost as the homeostatic tuning of neural thresholds. The later, in particular, is directly dependent on the level of the incoming noise in a non-monotonous fashion. In the absence of homeostatic tuning of neural thresholds (i.e., with zero linear cost), the predictive coding network shows regular Up states. We observe that the regularity of Up states is in fact due to the recovery of adaptive currents after an Up state. Similar dependency has been recently reported in [44], where the regularity of Up states is modulated by the extracellular level of potassium.

Slow currents (i.e. with a slower time scale than the fast network interactions required by predictive coding), as for example the NMDA current, were shown to have important contribution to quiescent state activity [45]. Introduced in a predictive coding network model, slow currents could implement a dynamical computation rather than just tracking of the input signals [14]. The present work describes a simplistic model, which captures only fast currents. Slow currents can be included in the model to perform other types of computation than signal tracking, for example differentiation of the input signal. Our future work is aimed at studying the interplay of fast and slow currents during spontaneous activity. In addition, it might be interesting to introduce heterogeneity in synaptic delays and in cost parameters. For simplicity, we implemented costs as static parameters, affecting equally all cells of the network. It is however closer to biology to assume costs as being variable over time and affecting different cells in a heterogeneous manner.

The mechanism that drives the activity in the quiescent state gives general insights into the network dynamics generated by recurrent connections. The mechanism that underlies quiescent state activity is obviously present also in the active state. In the active state, the observed activity of the network is a combination of internal dynamics and the stimulus driven response. A previous study [3] showed that the response of the network in the active state can be decomposed into spontaneous activity and stimulus-related response. The same study also shows that stimulus-induced part of the response is more or less invariant from one trial to another while the spontaneous part is variable. According to this scenario, it is the spontaneous part of the response that accounts for the trial-to-trial variability that is observed in the overall response. The stimulus-driven part of the response can therefore be seen as an invariant drive, kicking the network while it is going through continuously evolving internal dynamics. Our model corroborates same conclusions.

This work extends previous results that E/I balance is the dominant intrinsic source of single neuron variability [10, 46, 47]. From the perspective of the model with predictive coding, this variability is not a form of neural noise, but can be interpreted as a deterministic and chaotic neural code [14]. Present work shows that the model with predictive coding is robust to perturbations with purely stochastic processes when cost parameters are adjusted, both in terms of coding and in terms of dynamics. Similarly to [10], the model with predictive coding assumes excitatory-inhibitory balance of inputs to single neurons and displays chaotic dynamics, but, in contrast to the later, it assumes dense connectivity instead of sparse connections, which, locally at least, is closer to experimental observations [13, 48]. This stronger and denser connections allow for a larger repertoire of intrinsic dynamics, such as Up states, which can co-exist with the representation of input signals.

In the model with predictive coding, Up states are due to small perturbations, a single noise-induced spike, amplified by the strong excitatory recurrent connections in the network, and quickly corrected by a re-balancing through the activation of the inhibitory connections. When the network consists of more than 2 neurons, the network dynamics is chaotic and the timing of spikes from one perturbation to another is not reproducible [14]. Our prediction is compatible with the observation that small perturbations can induce a strong temporary increase in the population firing rate [49]. However, even if we propose a similar mechanistic interpretations than theirs, our interpretation in terms of implications for neural coding are radically different. The study [49] took the irreproducibility of spike timing and chaotic network dynamics as a support for rate coding. Our approach, in contrast, speaks in favor of a temporal code, where the timing of each spike matters and is determined by a precise computation, carried out by the network. In our case, the variability of spike patterns can be accounted for by the degeneracy of the neural code. Degeneracy, a source of intrinsic variability, is due to the mapping of low dimensional space of input variables to high dimensional space of network configurations. If the number of input variables is much smaller than the number of neurons, the resulting spiking pattern following the perturbation of a single neuron is determined by the specific configuration of the network at the moment of perturbation (i.e. the initial conditions). Interestingly, our approach also shows that the variability on the level of single units does not prevent an almost deterministic code at the population level. The high dimensionality of the representational space of the network allows for a multitude of spiking patterns that are nearly identically decoded. In such a context of a redundant neural code, trial-to-trial variability can be seen as sampling in the space of possible solutions for the same computational problems.

## Supporting Information

S1 Table List of variables and tables of network parameters. List of variables that define models with predictive coding and tables of parameters used for simulations.
(PDF)
