# Supplementary material for: Computational Account of Spontaneous Activity as a Signature of Predictive Coding
Source: PLoS Comput Biol. 2017 Jan 23;13(1):e1005355. doi: 10.1371/journal.pcbi.1005355 (PMC5293286; doi:10.1371/journal.pcbi.1005355)
Supplement: S1 Table — List of variables that define models with predictive coding and tables of parameters used for simulations. (PDF) [file pcbi.1005355.s001.pdf]

Supplementary information

Table 1 lists all variables that are part of the framework. Tables 2 and 3 list parameters that were used for simulations of the model with all-to-all connectivity and of the model with local connectivity, respectively. Parameters used for generating figure 1 are listed in the figure caption. Unless otherwise reported in the figure caption, we used fixed set of parameters, according to tables 2 and 3.

Table 1: List of variables

variable	description
$s_j(t)$	j-th input
$\bar{s}_j(t)$	j-th dimension of the signal
$\hat{s}_j(t)$	j-th dimension of the estimate
$V_i(t)$	membrane potential of neuron i
$r_i(t)$	instantaneous firing rate of neuron i
$o_i(t)$	spike train of neuron i
Thres_i	firing threshold of neuron i
$MUA(t)$	multi-unit activity
$E(t)$	objective function
$\langle error \rangle$	mean error
$\langle cost \rangle$	mean cost
$Total\ error = \alpha\langle eror \rangle + \beta\langle cost \rangle$	inverse efficiency

Table 2: Parameters of all-to-all connected network with random weights

parameter	description	value
J	number of inputs	3
N	number of neurons	400
λ	membrane time constant	4 Hz
w_{ij}	neural weights	$\mathcal{N}(0,1)$
λ_{input}	time constant of the input current	20 Hz
ν	linear cost constant	5
μ	quadratic cost constant	5
σ	standard deviation of the noise in the membrane potential	0.25 ms^{-1}
p_{spike}	probability of spiking when the threshold is reached	1
λ_D	time constant of the multi-unit activity	50 Hz
u.c.	Up state criterion	20 % of neurons simultaneously active
Δ	transmission delay	1 ms

Table 3: Parameters of spatially organized network with local connectivity

parameter	description	value
J	number of inputs	60
N	number of neurons	400
λ	membrane time constant	4 Hz
A	controls the strength of the input	0.3
B	controls the spread of the input	1
C	peak amplitude of the tuning curve	1
D	controls the width of the tuning curve	50
λ_{input}	time constant of the input current	2 Hz
ν	linear cost constant	19
μ	quadratic cost constant	19
σ	standard deviation of the noise in the membrane potential	0.1 ms^{-1}
p_{spike}	probability of spiking when the threshold is reached	1
λ_D	time constant of the multi-unit activity	50 Hz
u.c.	Up state criterion	20 % of neurons simultaneously active
Δ	transmission delay	2 ms
